# A Review of Gas-Sensitive Materials for Lithium-Ion Battery Thermal Runaway Monitoring

**DOI:** 10.3390/molecules31020347

**Published:** 2026-01-19

**Authors:** Jian Zhang, Zhili Li, Lei Huang

**Affiliations:** 1 Shanghai Key Laboratory of Chips and Systems for Intelligent Connected Vehicle, Shanghai 200444, China; 2 Research Center of Nano Science and Technology, College of Sciences, Shanghai University, Shanghai 200444, China; 3 School of Chemistry and Chemical Engineering, Guangxi University, Nanning 530004, China

**Keywords:** lithium-ion battery, TR warning, gas sensing, electrolyte vapor, H_2_, CO, CO_2_

## Abstract

Lithium-ion batteries (LIBs) face the safety hazard of thermal runaway (TR). Gas-sensing-based monitoring is one of the viable warning approaches for batteries during operation, and TR warning using semiconductor gas sensors has garnered widespread attention. This review presents a comprehensive analysis of the latest advances in this field. It details the gas release characteristics during the TR failure process and identifies H_2_, electrolyte vapor, CO, CO_2_, and CH_4_ as effective TR warning markers. The core of this review lies in an in-depth critical analysis of gas-sensing materials designed for these target gases, systematically summarizing the design, performance, and application research of semiconductor gas-sensing materials for each aforementioned gas in battery monitoring. We further summarize the current challenges of this technology and provide an outlook on future development directions of gas-sensing materials, including improved selectivity, integration, and intelligent advancement. This review aims to provide a roadmap that directs the rational design of next-generation sensing materials and fast-tracks the implementation of gas-sensing technology for enhanced battery safety.

## 1. Introduction

Lithium-ion batteries (LIBs) offer several advantages, including high specific energy, high specific power, low self-discharge rates, and long cycle life [1,2]. With the continuous optimization and improvement of battery performance, LIBs have been widely used as energy storage and power supply units in important fields, including transportation, portable electronic products, chemical energy storage systems, chemical production, and aerospace systems [3,4]. However, due to the influence of application environments and usage methods, LIBs may experience thermal runaway when an internal short circuit occurs or when they are subjected to abuse. This leads to a sharp rise in battery temperature, triggering accidents such as fires and even explosions, which pose a great threat to personal and property safety [5,6]. Therefore, improving the warning capability of LIBs when TR occurs and enhancing the safety of LIBs have received considerable attention in recent years [7].

To address the safety issues of LIBs, many researchers have focused on studying LIB TR. By investigating the occurrence process and mechanism of TR, they aim to develop targeted and reliable battery safety monitoring and TR warning technologies [8]. Studies have shown that when TR occurs in LIBs, it is accompanied by abnormal phenomena, including temperature rise, fluctuations in internal electrical signals (e.g., voltage, current, impedance), massive gas generation, and pressure increase. Integrating sensor technology with battery systems enables the identification and capture of changes in these abnormal signals, thereby providing warnings for potential battery faults [9,10]. In recent years, technologies for LIB TR warnings have developed rapidly, with research and applications emerging for various warning techniques. Metal–Oxide–Semiconductor (MOS) gas sensors are critically important for TR warning in batteries. Unlike temperature-based methods, which are hindered by measurement lag, or electrical signature monitoring, which requires complex and costly models, gas detection offers the earliest possible warning [8,11,12,13]. Characteristic gases are released before significant voltage or temperature changes occur. Among gas-sensing technologies, MOS sensors provide a uniquely practical solution due to their low cost, proven reliability, fast response (on the order of seconds), high sensitivity (down to ppb levels), and miniaturized form factor suitable for integration into battery systems. Research confirms their durability in harsh conditions and that strategic placement ensures effective warning without sensor damage. Their ability to deliver timely, reliable, and cost-effective monitoring makes MOS-based gas sensing a superior and essential pathway for proactive battery safety management [12,14].

While previous reviews have laid groundwork for gas-based TR monitoring, distinct gaps remain, particularly regarding a dedicated and systematic analysis of semiconductor sensing materials. For instance, Wang et al. [11] compared various monitoring methods such as acoustic, optical, thermal, mechanical, and electrical signals, but did not delve into sensor materials. Qian et al. [15] reviewed various advanced gas detection and warning technologies, but focused primarily on optical fiber sensing technologies. Although Teng et al. [16] and Shao et al. [17] provided more targeted reviews on semiconductor sensors for key TR gases, their discussions either lacked depth in connecting material properties to application performance or did not systematically cover the critical area of electrolyte vapor detection. Notably, a comprehensive review that specifically targets semiconductor gas-sensing materials for major TR markers, with explicit emphasis on electrolyte vapors and a clear “material–property–application” framework, remains absent.

Therefore, this article focuses on the latest research progress on semiconductor gas-sensing materials. First, we systematically introduce the composition of lithium-ion batteries and the different stages of TR, and briefly outline the gas generation characteristics during TR. Second, since there is some controversy in many existing studies regarding the selection of warning gases, we discuss the gas generation process and identify five key gases during TR, including electrolyte vapor, H_2_, CO, CO_2_, and CH_4_. Subsequently, we provide a comprehensive review of the research progress of gas-sensing materials used for TR warning. Finally, we present our views on the current applications of these materials and propose potential directions for future research.

## 2. Introduction to Lithium-Ion Batteries and TR

### 2.1. Introduction to Lithium-Ion Batteries

**Figure 1:**
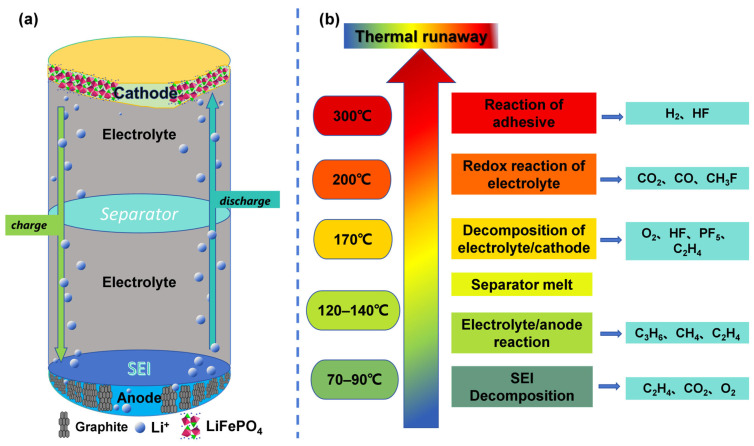
(**a**) Schematic diagram of battery structure and charge–discharge process; (**b**) schematic diagram of TR process.

The structure of a LIB is illustrated in Figure 1a. Typically, an LIB consists of a negative electrode, a positive electrode, an electrolyte, and a separator [18]. Positive electrode materials are lithium-containing compounds, such as lithium cobalt oxide (LCO), lithium iron phosphate (LFP), lithium manganese oxide (LMO), and ternary materials including lithium nickel cobalt manganese oxide (NCM) and lithium nickel cobalt aluminum oxide (NCA) [19,20]. Negative electrode materials require low potential and high reducibility [19], with graphite-based carbon materials being the most widely used [21,22]. A porous insulating separator is placed between the positive and negative electrodes. Common materials for separators include single-layer polyethylene (PE) or polypropylene (PP) films, PP/PE/PP three-layer composite films, and ceramic-coated separators with heat resistance and dendrite suppression properties [23]. The key function of the separator is to block electron conduction and prevent internal short circuits of the battery, while ensuring the free transport of lithium ions, making it crucial for both battery safety and performance [24]. The electrolyte is composed of lithium salts (e.g., the mainstream LiPF_6_), mixed carbonate solvents (e.g., dimethyl carbonate (DMC), diethyl carbonate (DEC), dimethyl ether (DME), ethyl methyl carbonate (EMC), ethylene carbonate (EC), and propylene carbonate (PC)), and functional additives [19,25,26]. Additionally, a film forms on the surface of the negative electrode, known as the SEI film [27]. It is a passive film generated during the first charging cycle of the battery, resulting from the reaction between the electrolyte and lithium intercalated in the negative electrode. The solute LiPF_6_ can decompose to form LiF, while the solvent can react with Li to generate substances such as Li_2_CO_3_ and ROCO_2_Li [28,29,30]. These products collectively form a film with a dense inner layer and a loose outer layer on the surface of the negative electrode [31]. The solid electrolyte interphase (SEI) film can effectively block the passage of electrons while allowing lithium ions to intercalate and deintercalate freely into and from the negative electrode [32]. After formation, the film acts as a barrier to prevent the continuous reaction between the electrolyte and the negative electrode, reducing the consumption of active lithium and irreversible capacity loss [33]. Additionally, it can buffer the volume change of the negative electrode during charge–discharge cycles (e.g., according to A. Mukhopadhyay et al. [34], graphite undergoes a volume expansion of approximately 14% after lithium intercalation) and protect the electrode structure [35,36].

The normal charge–discharge process of an LIB is illustrated in Figure 1a. Taking a lithium iron phosphate (LiFePO_4_) positive electrode and a graphite negative electrode as examples:

During charging, an oxidation reaction occurs at the positive electrode, while a reduction reaction takes place at the negative electrode. Lithium ions in the LiFePO_4_ crystal deintercalate from the positive electrode and are driven to migrate through the electrolyte and separator to the negative electrode. Simultaneously, ferrous ions are oxidized, and LiFePO_4_ in the positive electrode material is converted to FePO_4_. At the negative electrode, the migrated Li^+^ can penetrate the SEI film, gain electrons on the negative electrode surface, and intercalate between graphite layers to form the lithium–carbon compound LiC_6_. During discharging, the battery acts as a power source to supply electricity externally, and the reverse of the above process occurs. Li^+^ deintercalates from the negative electrode, returns to and intercalates into the positive electrode via the electrolyte and separator, while electrons flow from the negative electrode to the positive electrode through the external circuit to drive the load [12].

### 2.2. Introduction to TR

According to literature reports, lithium-ion batteries exhibit multiple synergistic degradation pathways [37,38,39]:

1. Loss of active lithium and increase in internal resistance: Based on Failure Modes, Mechanisms, and Effects Analysis (FMMEA), reduction reactions and deposition at the anode may induce lithium plating, excessive thickening of the SEI layer [40], and accumulation of by-products, which irreversibly consume active lithium and lead to capacity fading. Additionally, the thickening of the SEI layer exacerbates interfacial impedance and internal resistance, resulting in power degradation, while the decomposition of the SEI layer at elevated temperatures may trigger TR.
2. Degradation during battery cycling: Abnormal charging/discharging processes (e.g., fast charging, overcharging, or over-discharging) can disrupt the equilibrium of lithium ions intercalation/deintercalation, inducing polarization and internal stress. This causes cracking of electrode particles and damage to the conductive network, leading to the loss of electrical contact and an increase in ohmic resistance. The exposed fresh surfaces further form new SEI layers, which exacerbates capacity and power fading.
3. Internal Short Circuit (ISC): ISC generally refers to the formation of a conductive path between the cathode and anode due to the failure or destruction of the internal separator. This results in intensified self-heating and local overheating, ultimately triggering TR. ISC is often caused by the growth of lithium dendrites, particle contamination, or mechanical compression/puncture of the separator. Model-based or data-based warning methods for ISC can capture characteristics induced by ISC, such as impedance variations, state of charge (SOC) discrepancies, and internal voltage anomalies, through electro-thermal coupling models, data-driven models (e.g., LSTM, KPCA), and other approaches [41,42]. Thus, they exhibit certain advantages in ISC monitoring.

These degradation mechanisms may occur simultaneously, interacting with and accelerating each other. They weaken battery stability, and their continuous evolution will ultimately lead to TR events. LIBs may experience these failures due to internal/external factors or abusive conditions. If the temperature rises continuously, it will trigger thermal decomposition of internal materials and exothermic side reactions, eventually leading to uncontrolled temperature increase. When the temperature rise rate dT/dt exceeds 1 °C/s, the self-heating rate accelerates, which is widely recognized in the literature as the onset of TR. Without timely warning and intervention, this will result in severe consequences such as fire and explosion [43,44].

The triggers of TR mainly fall into three categories [13,45]: (1) Mechanical abuse, such as battery deformation caused by crushing, puncturing, or impact; (2) Electrical abuse, such as abnormal usage including overcharging, over-discharging, or internal short circuit; (3) Thermal abuse, such as exposure to high-temperature environments or local overheating. Any abuse beyond a certain threshold will damage battery stability and initiate TR. Studies have shown that the TR of LIBs typically proceeds through the following period.

#### 2.2.1. Decomposition of the SEI Film on the Negative Electrode Surface

The SEI film on the negative electrode surface is considered the most vulnerable component of LIBs at elevated temperatures [46]. This SEI film contains some metastable substances, including ROCO_2_Li, (CH_2_OCO_2_Li)_2_, ROLi, and oxygen-containing polymers, which are prone to decomposition at higher temperatures [28].

When the battery temperature reaches 70–90 °C, the SEI film begins to decompose first. The temperature range exhibits some fluctuation and largely depends on environmental conditions and measurement methods; for example, the study by Wang et al. [47] showed that decomposition starts at 57 °C. As the temperature approaches 90 °C, the exothermic effect of the SEI film decomposition reaction becomes significant. The reactions involved are as follows [48]:

(1) $${\left(\mathrm{ROCOOLi}\right)}_{2}\;\to \;{\mathrm{Li}}_{2}{\mathrm{CO}}_{3}\;+\;C_{2}H_{4}↑\;+{\;\mathrm{CO}}_{2}↑\;+\;0.5O_{2}↑$$

In addition, the lithium that migrates and intercalates into the graphite negative electrode may react with certain species in the SEI film to generate Li_2_CO_3_ and C_2_H_4_. The reaction process is as follows:

(2) $$2\mathrm{Li}\;+\;({\mathrm{CH}}_{2}\mathrm{OCOLi}{)}_{2}\;\to \;2{\mathrm{Li}}_{2}{\mathrm{CO}}_{3}\;+\;C_{2}H_{4}↑$$

The above reactions release gases including CO_2_, C_2_H_4_, and O_2_. Among these, O_2_ acts as a strong oxidizing agent and accelerator. Both the O_2_ generated at this stage and the O_2_ produced during the thermal decomposition of the positive electrode material (discussed later) will intensify the exothermic reactions inside the battery, potentially endangering battery performance and safety [28].

It should be noted that batteries typically do not exhibit venting behavior at this stage, making monitoring relatively challenging. However, if micro-sensors can be embedded inside the battery to detect gases generated during this phase (e.g., C_2_H_4_, CO_2_), ultra-early warning can be achieved as early as the stage of abnormal decomposition of the SEI layer.

#### 2.2.2. Reactions Between Lithium in the Negative Electrode and Electrolyte Solvents

As the internal temperature of the battery continues to rise, when it reaches 120–140 °C, the SEI layer decomposes almost completely [49]. Without the protection of the SEI layer, the negative electrode comes into direct contact with the electrolyte. Metallic lithium intercalated in the graphite negative electrode then reacts exothermically with organic solvents in the electrolyte, such as EC, PC, and DMC, and generates gases. The main reactions are as follows [50,51]:

(3) $$2\mathrm{Li}\;+\;C_{3}H_{4}O_{3}(\mathrm{EC})\;\to {\;\mathrm{Li}}_{2}{\mathrm{CO}}_{3}\;+{\;C}_{2}H_{4}↑$$

(4) $$2\mathrm{Li}+{\;C}_{4}H_{6}O_{3}(\mathrm{PC})\;\to \;{\mathrm{Li}}_{2}{\mathrm{CO}}_{3}+C_{3}H_{6}↑$$

(5) $$2\mathrm{Li}+{\;C}_{3}H_{6}O_{3}(\mathrm{DMC})\;\to \;{\mathrm{Li}}_{2}{\mathrm{CO}}_{3}+{\;C}_{2}H_{6}↑$$

(6) $$2\mathrm{Li}+C_{5}H_{10}O_{3}(\mathrm{DEC})\;\to \;{\mathrm{Li}}_{2}{\mathrm{CO}}_{3}+{\;C}_{2}H_{4}↑+{\;C}_{2}H_{6}↑$$

These processes are actually the regeneration of the SEI film. As the temperature continues to rise, however, these regenerated SEI films become unstable and lose their ability to protect the metallic lithium intercalated in the negative electrode. The chain reactions between the negative electrode and the electrolyte thus persist [28,45,48]. This process is characterized by several distinct features: the release of gases (e.g., C_2_H_4_, C_3_H_6_), slight battery swelling, mild heat generation from the reactions, and a gradual increase in temperature, with no significant changes in voltage or current.

As presented above, the gases generated at this stage are primarily composed of hydrocarbons. If these gases, along with the CO_2_ from the previous stage, can be detected at this phase, early warning can be achieved before a more severe temperature rise occurs.

#### 2.2.3. Separator Failure and Thermal Oxidation Reactions

As the internal temperature of the battery continues to rise, the separator material will reach its melting point and eventually shrink or melt, specifically at about 135 °C for PE, 166 °C for PP, and 200 °C for ceramic separators. This makes the positive and negative electrodes come into direct contact, triggering an internal short circuit. The heat generated then drives the battery’s internal temperature to surge sharply from approximately 120 °C to over 300 °C [48,52]. This process is also recognized as the transition of the failure from a controllable state to an uncontrollable one [50]. During this intense temperature rise, the active materials of the positive electrode, such as LiCoO_2_ and LiFePO_4_, also undergo high-temperature decomposition, releasing oxygen and heat. The oxygen produced then reacts with the organic solvents in the electrolyte (e.g., EC, DEC, DMC) in oxidation reactions, generating significant amounts of heat [53]. Thus, the thermal decomposition of the positive electrode material and the oxidation of the electrolyte are both recognized as the reaction stages in the TR process that generate the most heat and cause the most dramatic temperature rise [8]. The oxygen-releasing reactions of common positive electrode materials are as follows [8,54,55].

(7) $$2{\mathrm{LiFePO}}_{4}\;⟶\;{\mathrm{Fe}}_{2}P_{2}O_{7}+\frac{1}{2}O_{2}↑$$

(8) $${\mathrm{Li}}_{x}{\mathrm{CoO}}_{2}\;⟶\;x{\mathrm{LiCoO}}_{2}+\frac{1\text{-}x}{3}{\mathrm{Co}}_{3}O_{4}+\frac{1\text{-}x}{3}O_{2}↑$$

(9) $$Li_{x}Mn_{2}O_{4}\;\to \;\mathrm{xLiMn}O_{2}+\frac{x}{3}Mn_{3}O_{4}+(1\text{-}x)Mn_{2}O_{3}+\frac{3\text{-}x}{6}O_{2}↑$$

(10) $${\mathrm{Li}}_{0.35}(\mathrm{NiCoMn}{)}_{1/3}O_{2}\;⟶{\;\mathrm{Li}}_{0.35}(\mathrm{NiCoMn}{)}_{1/3}O_{2\text{-}y}+\frac{y}{2}O_{2}↑$$

(11) $$\mathrm{NCM}(R\bar{3}m)\;⟶\;(\mathrm{Mn},\mathrm{Ni})O(\mathrm{Fm}\bar{3}m)+\mathrm{CoO}+\mathrm{Ni}+{\;O}_{2}↑$$

During the above processes, a large amount of oxygen is generated alongside significant heat release. While the positive electrode material decomposes, LiPF_6_, which is the lithium salt serving as the electrolyte solute, also undergoes thermal decomposition [56,57]. Other side reactions occur at this stage as well, generating certain alkyl fluorides and hydrocarbons. In addition, when the internal temperature of the battery exceeds 200 °C, the O_2_ previously released from the decomposition of the positive electrode material will further participate in redox reactions with electrolyte solvents and graphite in the negative electrode [58,59].

(12) $${\mathrm{LiPF}}_{6}\;\to \;\mathrm{LiF}+{\mathrm{PF}}_{5}↑$$

(13) $$C_{3}H_{4}O_{3}{/C}_{4}H_{6}O_{3}/C_{3}H_{6}O_{3}+O_{2}\;\to \;{\mathrm{CO}}_{2}+\mathrm{CO}+H_{2}O$$

Moreover, the electrolyte solvents themselves decompose at high temperatures, producing large amounts of gases such as CO_2_ and H_2_. In summary, the various decomposition and oxidation reactions at this stage release a great deal of heat, causing the temperature to rise sharply and releasing gases like CO and CO_2_ [60,61,62,63]. Monitoring gases such as CO, CO_2_, and H_2_ at this stage can trigger an alarm for the damage of the battery separator and thermal oxidation reactions.

#### 2.2.4. Reactions of Binders

Finally, when the battery temperature exceeds 260 °C, binders such as polyvinylidene fluoride (PVDF) and carboxymethyl cellulose (CMC), which are used to bond electrode materials, conductive agents, and other components to maintain electrode integrity, undergo thermal decomposition or react with lithium leached from lithiated graphite. This process generates HF and large amounts of H_2_ [64].

(14) $$\text{-}{\mathrm{CH}}_{2}\text{-}{\mathrm{CF}}_{2}\text{-}\;\to \;\text{-}\mathrm{CH}=\mathrm{CF}\text{-}+\mathrm{HF}$$

(15) $$\text{-}{\mathrm{CH}}_{2}\text{-}{\mathrm{CF}}_{2}\text{-}+\mathrm{Li}\;\to \;\mathrm{LiF}+\text{-}\mathrm{CH}=\mathrm{CF}\text{-}+{0.5H}_{2}$$

(16) $$\mathrm{CMC}\text{-}\mathrm{OH}+\mathrm{Li}\;\to \;\mathrm{CMC}\text{-}\mathrm{OH}+0.5H_{2}$$

At this point, there is little time left before the battery triggers severe accidents such as smoke and fire. However, monitoring gases such as H_2_ and HF at this stage can still be used to issue the final alarm.

In summary, it can be observed that numerous complex reactions occur during the TR process of LIBs, producing gases such as O_2_, H_2_, carbon oxides (CO_2_, CO), hydrocarbons (C_2_H_4_, CH_4_, etc.), and fluorine-containing compounds (e.g., HF, CH_3_F, etc.). In Table 1, we have summarized the perniciousness of these gases. As the TR process proceeds, the electrolyte vapor in LIBs and the flammable gases produced by various decomposition reactions accumulate; this accumulation causes the pressure to rise rapidly. Since the gases cannot dissipate quickly, the battery first deforms and swells, and eventually, venting occurs [13,65]. A large amount of heat accumulates inside the battery, causing the internal temperature to rise rapidly. This eventually develops into a safety incident and may even lead to fires and explosions. Therefore, it is particularly important to develop effective warning methods for TR so that remedial measures can be taken as soon as possible.

## 3. Common Gases Venting from TR and Influencing Factors of Battery Venting

We have summarized the TR process; however, the reactions occurring during actual TR are more complex, involving numerous side reactions that generate a wide variety of gases. To date, numerous studies have explored the TR mechanisms and gas generation characteristics of different batteries in detail. The research methods typically involve inducing TR through abusive conditions such as heating, overcharging, or puncturing, followed by qualitative and quantitative analysis of the released gases using techniques like gas chromatography (GC) and Fourier-transform infrared spectroscopy (FT-IR).

Comprehensive analysis of multiple studies indicates that H_2_, CO, CO_2_, HF, CH_4_, C_2_H_6_, C_3_H_6_, C_3_H_8_, and C_4_H_10_ are the main gas products during TR [15,66,67]. Among these, CO_2_, H_2_, and CO account for the largest proportion of all gases, serving as the primary components of TR gases in lithium-ion batteries. In many cases, these three gases can even account for approximately 70% of the total gas production [67].

Nevertheless, variations exist in the specific quantity and proportion of each gas generated during TR between different types of batteries and between batteries of the same type but with different specifications. Such differences are typically influenced by factors including battery material types (such as electrode materials, separators, electrolytes, and fillers), operating environments, state of charge (SOC), and the type of TR initiation method (overcharging or heating) [15]. Below, we discuss and summarize several studies while highlighting the main types of TR gases.

Electrolyte composition and its decomposition behavior affect TR gas generation. Studies have shown that a significant portion of the gas produced during TR originates from the decomposition of organic solvents at specific temperatures [63,68], and this pyrolytic gas generation process becomes more intense with increasing temperature (with an acceleration turning point occurring at approximately 200 °C) [60]. Rong Da et al. [60] conducted pyrolysis experiments on electrolytes composed of LiPF_6_ and EC/DMC using an autoclave. They observed only 3 gases (CO, CO_2_, and C_3_H_6_) at 120 °C, whereas as many as 13 different gases were detected at 210 °C. Lamb et al. [61] investigated the decomposition and gas generation behavior of mixtures of EC, DEC, DMC, EMC, and LiPF_6_ at 400 °C, finding that all organic solvents produce large amounts of CO_2_ during thermal decomposition. Additionally, DEC generates substantial flammable gases (H_2_, C_2_H_6_, and C_3_H_8_), EC produces some H_2_, and EMC decomposition tends to generate C_2_H_6_, C_3_H_8_, and H_2_, while DMC is the most stable, producing a small total gas volume dominated by CO_2_ with minimal yields of other gases.

SOC affects the gas generation behavior of TR. SOC refers to the amount of electricity stored in a battery and is closely related to battery safety [69]. When a lithium-ion battery is abused, the higher its SOC, the more severe the internal side reactions [70]. A.W. Golubkov et al. [55] argued that batteries with different SOCs exhibit distinct gas generation characteristics during TR. They induced TR in NCM and LFP batteries via external heating, detected gases including CO_2_, H_2_, CO, CH_4_, C_2_H_4_, and C_2_H_6_ using gas chromatography, and calculated the quantity and proportion of each gas. Multiple experiments conducted under different SOC conditions confirmed that the total gas production of lithium-ion batteries during TR increases significantly with rising SOC. Meanwhile, the gas composition also changes: the proportion of CO_2_ decreases with increasing SOC, while that of H_2_ and CO shows an increasing trend. However, it should be noted that CO_2_ still accounts for the largest quantity among all gases in most cases.

**Figure 2:**
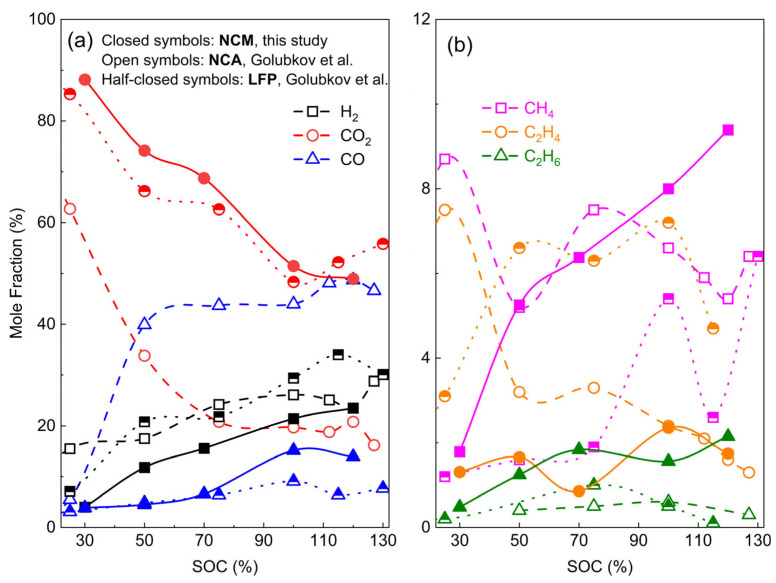
The generated gas compositions of NCM [71], NCA [55] and LFP [55] cells with different SOCs: (**a**) major components (H_2_/CO_2_/CO) and (**b**) minor components (CH_4_/C_2_H_4_/C_2_H_6_). In the figure, the closed symbols represent NMC, open symbols represent NCA, half-closed symbols represent LFP.

Zhang et al. [71] also investigated the gas generation characteristics of NCM batteries with different SOCs (120%, 100%, 70%, 50%, 30%) by inducing TR through external heating. Similar to the LFP batteries studied by Golubkov et al. [55], the NCM batteries in their research were dominated by CO_2_ in the generated gases, followed by H_2_ and CO. Based on this study and that of Golubkov et al. [55], they plotted a concentration–SOC graph for comparison, as shown in Figure 2. It can be seen that with increasing SOC, the concentration of CO_2_ decreases while that of H_2_ and CO tends to increase, which is consistent with the conclusions of the two studies.

In addition, different types of electrode materials also exert distinct impacts on the gas generation behavior during TR. This is most likely attributed to the differences in thermal stability among various cathode materials and their ability to participate in chemical reactions and release oxygen during TR. Studies have shown that the thermal stability of several common cathode materials follows the order: LMO > LFP > NCM > NCA > LCO, while the self-heating rate sequence is LFP < LMO < NCM < NCA < LCO [69].

Yuan et al. [66] induced TR by heating with an accelerating rate calorimeter (ARC) to study the TR behavior of lithium batteries with three different electrodes (NCM, LFP, and LTO). They analyzed the vent gases of different batteries using gas chromatography and summarized the main gases and their concentrations, as shown in Table 2.

It can be observed that the type of cathode material affects the vent gas concentrations. Specifically, the TR gas of LFP batteries is dominated by CO_2_ and H_2_, with other gases accounting for a very small proportion; LTO batteries are mainly composed of CO_2_, with low contents of H_2_ and CO, and even fewer other gases, while NCM batteries produce more CO during TR, followed by CO_2_, H_2_, and CH_4_. In addition, the proportion of most hydrocarbons is extremely low.

**Figure 3:**
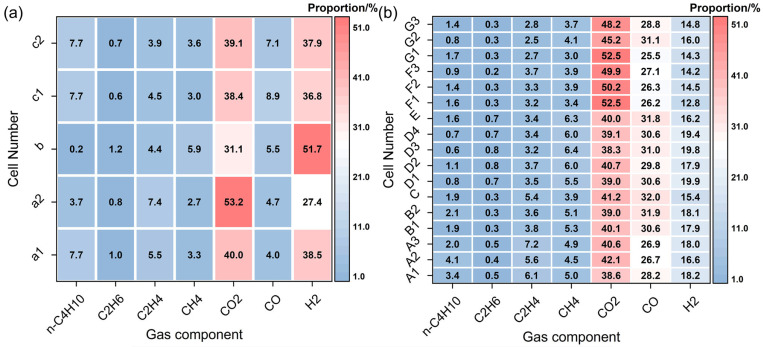
Proportion of various gases generated during TR: (**a**) LFP cells [54]; (**b**) NCM cells.

G Wei et al. [54] also reached similar conclusions in their research. They investigated the vent gases of four commercial lithium-ion batteries during TR, inducing TR via heating with an accelerating rate calorimeter (ARC) and identifying the battery vent gases (BVG) using gas chromatography. They confirmed that H_2_, CO, CO_2_, CH_4_, C_2_H_4_, C_2_H_6_, and n-C_4_H_10_ were present in the vent gases of all batteries; however, batteries with different electrode materials exhibited distinct gas generation characteristics, as shown in Figure 3a,b. In five TR tests of LFP batteries, H_2_ and CO_2_ accounted for the largest proportions of all generated gases and were present in comparable quantities, with H_2_ content ranging from 27% to 52% and CO_2_ from approximately 31% to 53%, while CO accounted for a small proportion of only 4% to 9%. In contrast, NCM batteries with a higher nickel content emitted gases with a higher proportion of carbon oxides: CO accounted for 25% to 32%, CO_2_ for 38% to 52%, and H_2_ volume fraction was only about 12% to 20%, significantly lower than that in LFP batteries. These results are consistent with the previous conclusions of Yuan et al. [66]. It should be noted that in this study, the authors stated that gases such as HF and electrolyte vapor were not included in the consideration. In addition, they found that LFP batteries exhibit greater stability at the cell level and pose lower thermal risks during the TR process, whereas NCM batteries with higher energy density show the opposite trend. Furthermore, the TR hazard of NCM batteries intensifies with increasing nickel content, characterized by higher maximum temperature (T_max_) and maximum temperature rise rate (R_max_), which is consistent with a study conducted by A.W. Golubkov et al. [67].

However, actually, besides the gases highlighted in the aforementioned studies, electrolyte vapor and fluorides are also generated during the TR process. Fernandes et al. [72] conducted a study on commercial LFP cells. Instead of inducing TR via heating, they triggered it through overcharge abuse testing, followed by the identification and quantification of battery vent gases using FTIR. They confirmed that in addition to small-molecule gases (e.g., CO_2_, H_2_, HF, CH_4_, C_2_H_4_), a large amount of electrolyte solvent vapors (e.g., DMC and EMC) were generated and released. Additionally, several fluorides (HF, CH_3_F) were also detected. Depeng, K et al. [13] summarized the vent gases from abuse tests based on various studies, pointing out that the main gas products of TR include H_2_, CO, CO_2_, and other small-molecule gases, as well as some volatile organic compounds (VOCs). They further confirmed that electrolyte solvents are the primary component of battery vent gases under non-combustion conditions (e.g., the first venting event), which is consistent with the aforementioned study.

In summary, these studies have shown that during actual TR, the gas generation characteristics and venting gas concentration of batteries vary due to factors such as battery differences (specifications, types, state of charge) and TR triggering methods [55,61,70,73]. Nevertheless, the main types of gases from battery venting remain consistent. It can be confirmed that the vented gas is mainly composed of a considerable amount of electrolyte vapor (e.g., DMC, EMC, DEC [13,72]), as well as CO_2_, CO, and H_2_ [55,67], along with small amounts of small-molecule hydrocarbons (e.g., CH_4_, C_2_H_4_, C_2_H_6_), fluorides (e.g., HF, C_2_H_5_F, PF_5_), and O_2_ [11,15,66,67].

## 4. Optimization of Target Gases for TR Monitoring

However, not all these gases are suitable as target gases for TR monitoring. For example, regarding O_2_ and HF, although O_2_ is also released during TR, its content is low, and it is unstable, prone to reacting to form other gases. Additionally, since high concentrations of O_2_ are already present in the air, this can easily introduce interference during monitoring. For this reason, relying solely on O_2_ detection may compromise the accuracy of TR warnings [11,45]. In addition, research on the detection of fluorides released during the TR of batteries is also limited. This is because the concentration of these fluorides is low, and as a highly toxic gas, HF poses certain risks and challenges for gas-sensing research [45]. Thus, it is difficult for HF to become a universal warning gas. Therefore, neither O_2_ nor HF is the preferred warning gas for monitoring TR. However, there are still some differing views among different studies regarding the selection of preferred target gases, with the main focus on which gas is generated in the early stage of TR [74,75,76].

Some studies suggest that H_2_ can be used for TR warning [17,45]. H_2_ typically accounts for a relatively high proportion of TR gases; in addition to being produced by high-temperature side reactions in the late stage of TR [11], some studies have found that a certain amount of H_2_ also appears during the lithium dendrite growth stage [74]. During battery charging, lithium ions deintercalate from the cathode and intercalate into the graphite anode. However, uneven lithium deposition on the anode occurs under abuse conditions or abnormal charging (e.g., overcharging, fast charging), and this leads to the excessive growth of lithium dendrites [31,77,78]. On one hand, this excessive growth pierces the separator and triggers internal short circuits and side reactions; on the other hand, the lithium dendrites themselves react to generate H_2_, which becomes one of the sources of this gas.

**Figure 4:**
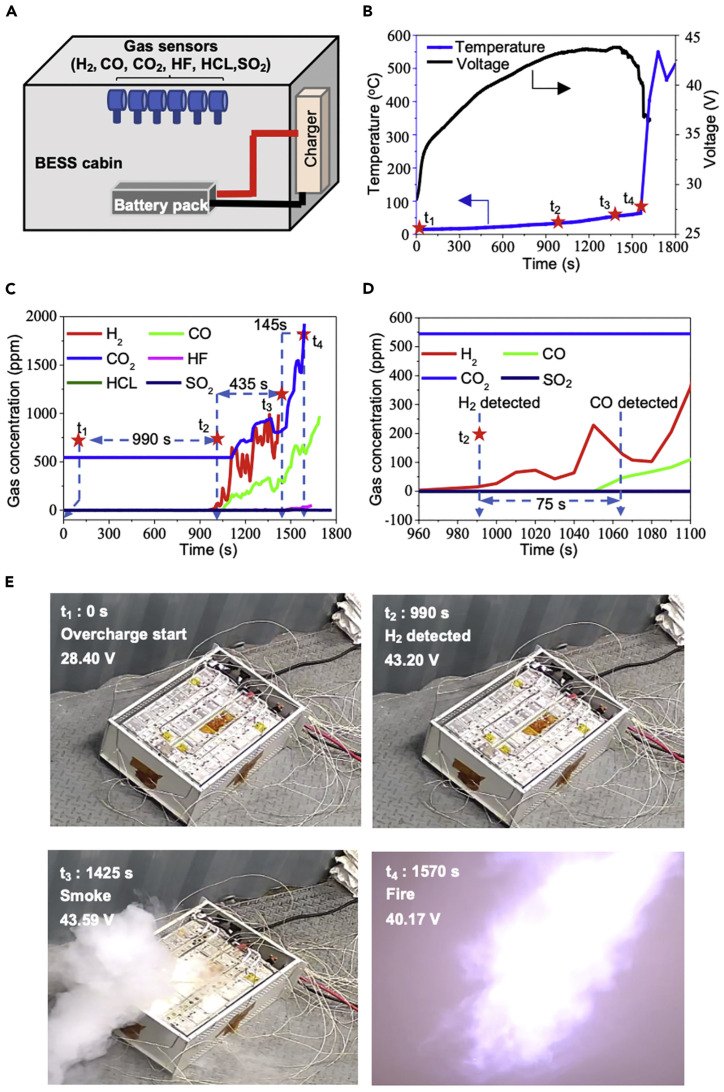
Overcharge Experiment of a LiFePO_4_ Battery Pack (8.8 kWh) with Online Detection of Six Gases [74]. (**A**) Schematic diagram of the real BESS cabin experimental environment. Six gas sensors (H_2_, CO, CO_2_, HF, HCl, and SO_2_) are installed above the battery pack. (**B**) Voltage distribution and surface temperature changes of the LiFePO_4_ battery pack during overcharge (charging current: 0.5 C). (**C**) Gas concentration changes of the six gases from 0–1800 s. (**D**) Gas concentration curves enlarged from 960–1100 s. (**E**) Optical images of LiFePO_4_ battery packs at different times. t_1_ = 0 s, initial overload time; t_2_ = 990 s, H_2_ gas detected; t_3_ = 1425 s, smoke appears; t_4_ = 1570 s, fire and explosion.

In 2020, Jin et al. [74] confirmed that overcharging abuse leads to the growth of micrometer-scale lithium dendrites on the graphite anode, through battery overcharging abuse experiments and in situ tests, as shown in Figure 4. They also found that H_2_ originates from the spontaneous reaction between Li metal dendrites and common electrode binders. They developed a method for TR detection by capturing H_2_, and confirmed that the H_2_ sensor triggers an alarm more promptly. It is 639 s earlier than smoke, 769 s earlier than fire, and also captured earlier than other gases (e.g., CO, CO_2_) detected online in the experiment. They further proposed using H_2_ as an early monitoring target: by monitoring the growth of lithium dendrites with an H_2_ sensor, it is possible to effectively provide a warning of TR.

**Figure 5:**
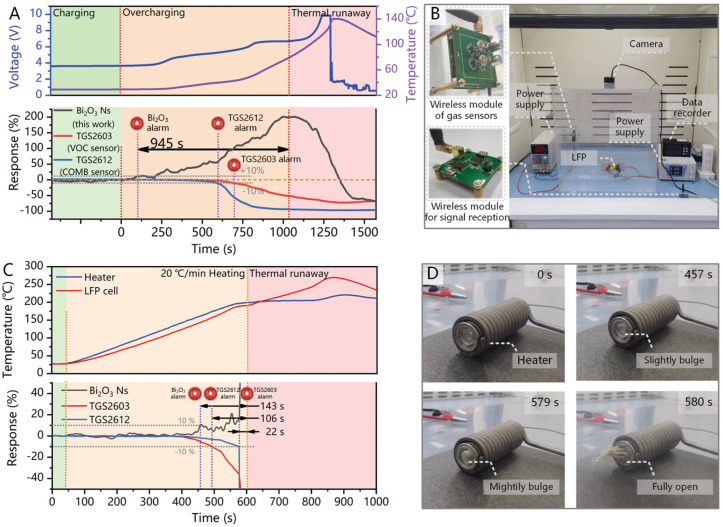
Testing the warning performance of different gas sensors for TR in LFP batteries [75]: (**A**) Warning performance of voltage/temperature sensor, Bi_2_O_3_-based DMC sensor, commercial VOCs sensor and combustible (COMB) gas sensor during overcharge testing. (**B**) Device used for overload simulation. (**C**) Condition of the LFP battery and the warning performance of different sensors during overheating tests. (**D**) LFP images at critical moments of the overheating test.

Some studies, however, argue that electrolyte vapor is a more favorable choice as a warning gas, as it emerges much earlier than other gases and exhibits an extremely high concentration [13,28,75]. For instance, Pan et al. [75] pointed out that DMC is a key component of the electrolyte in LIBs and that it evaporates when heated in the early stage of TR. Through battery overcharging-induced TR experiments, they verified that DMC serves as a critical marker released in the early phase of TR. The experimental setup is shown in Figure 5. After fully charging the batteries, they initiated the overcharge experiment and recorded the time elapsed after overcharging. They divided the TR process into three stages and confirmed that the expansion and vaporization of DMC occur in the early part of the first stage (at 390 s after overcharge starts), which is detectable earlier than traditional gases such as CO (482 s), CH_4_ (738 s), and H_2_ (1312 s). Additionally, the detectable concentration of DMC is much higher than that of other gases, and when the safety valve is fully opened, a high concentration of DMC (nearly 1000 ppm) can be detected. Nevertheless, gaseous DMC has poor diffusivity and cannot spread over long distances, as electrolyte vapor tends to condense on the surfaces of nearby objects or chamber walls when cooled. This condensation may cause fluctuations in DMC concentration, which explains the slight rise and fall observed in the sensor response at the initial stage. However, as TR progresses, both the temperature and the concentration of vented gas increase further, leading to a steady upward trend in the sensor response. What is more, the team conducted overheating experiments and external short-circuit tests, which verified the potential of the developed DMC sensor for TR warning applications. Thus, it can be confirmed that electrolyte vapor is an effective warning gas for LIB TR.

In addition, the main organic solvents in electrolytes typically include DMC, DEC, DME, EMC, EC, and PC [79]. Due to differences in volatility, the ease of monitoring varies among different electrolytes. Fernandes et al. [80] conducted thermal decomposition analysis experiments on LIB electrolyte solvents: they calculated the composition of a mixed solvent containing four carbonates (DMC, EMC, EC, and PC) and found that at temperatures of 180 °C, 240 °C, and 300 °C, EC and PC accounted for only 0.7%, 1.3%, and 2.0% of the gas phase, respectively. This is because both EC and PC are cyclic carbonates, with boiling points much higher than those of linear carbonate solvents (DMC, EMC, DEC) and linear ether (DME). As a result, they generally account for a low proportion of the electrolyte vapor. Therefore, when considering electrolyte vapor as a warning gas for TR, most studies select low-boiling-point chain organic solvents such as DMC, DEC, EMC, and DME.

**Figure 6:**
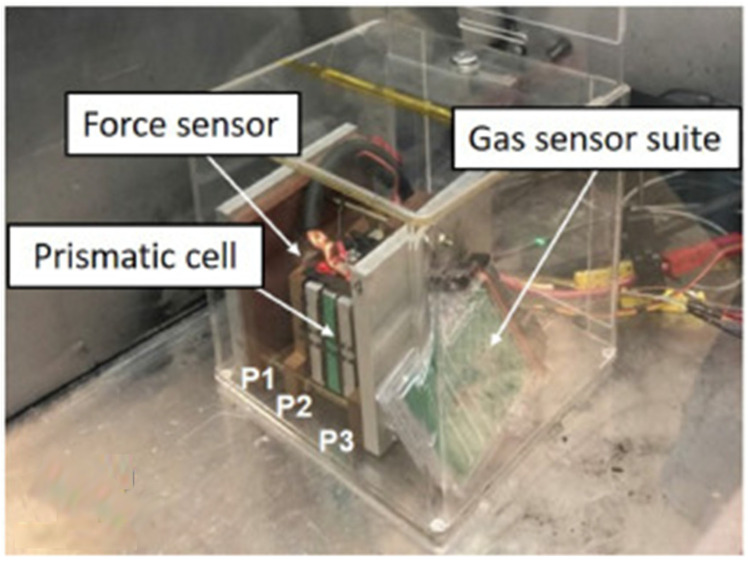
The overcharging experimental setup. The fixture was placed in an unsealed enclosure with a prototype gas sensor suite by Amphenol Advanced Sensors, which measures the CO_2_ concentration, humidity and gas temperature [76].

However, Cai et al. [76] hold different opinions. They summarized studies on TR gas emission, and CO_2_, CO, H_2_, and volatile organic compounds (VOCs, including electrolyte vapors) were identified as viable for TR warning and pointed out that CO_2_ is an ideal monitoring target because it is released early under all abuse conditions and has a relatively high concentration. Innovatively, they applied non-dispersive infrared (NDIR) sensors to CO_2_ monitoring during TR. They assembled the experimental device as shown in Figure 6. Overcharge experiments of NMC prismatic cells showed that the sensor had a fast response speed: it detected a CO_2_ concentration exceeding 10,000 ppm within 11 s after the battery exhausted gas, which confirms its feasibility. However, a certain concentration of CO_2_ inherently exists in the air, which may cause false alarms. Therefore, a reasonable detection threshold needs to be set. Through calculations, they set the detection threshold for the battery used in this experiment at 238,000 ppm, which can both avoid false alarms and maintain good detection capability. In addition to the above studies, some researchers have also investigated two other gases, namely CO and CH_4_, and concluded that they can also serve as effective characteristic gases for TR detection [81].

As demonstrated by the above studies, researchers differ in their considerations of characteristic gases. Cai et al. [76] identified their target gas according to its consistent presence and early release characteristics, while Jin et al. [74] did not include electrolyte vapor in the category of characteristic gases, a situation also found in the work of some other researchers. For example, when investigating the TR gas emission of four types of commercial batteries, Wei et al. [54] mentioned that gases such as HF and electrolyte vapor were not within the scope of their study, which is likely due to the poor diffusivity of electrolyte vapor and its susceptibility to combustion [13,76]. Furthermore, different researchers have obtained different sequences of gas generation. For instance, both [74,75] induced TR through overcharging, yet their conclusions regarding the generation sequence of various gases differed, and the emission sequence of H_2_ was even completely opposite. We speculate that these differences may stem from the following factors:

1. Battery differences. Pan et al. used a single 18650-type LiFeO_4_ battery, while Jin et al. used commercial LiFeO_4_ battery packs (including prismatic and pouch batteries). Due to the smaller capacity of 18650-type batteries, it may be difficult to generate a sufficient amount of lithium dendrites to participate in the reaction and produce H_2_ in the early stage of overcharging. On the other hand, even if H_2_ is generated, it may be undetectable because its content is below the detection limit. It is not until the late stage of TR (at approximately 200 °C [60]) that a large amount of electrolyte undergoes pyrolysis [62,63,68,82], and the intercalated Li in the negative electrode reacts with the binder [13], leading to the generation of a large quantity of H_2_ that can be detected.
2. Differences in overcharging time and battery venting time. In study [74], the interval from the start of overcharging to battery venting was 971 s, whereas in [75], this interval was only 324 s. Obviously, the overcharging time of the batteries in [74] was much longer than that in [75]. Overcharging time may reflect the extent to which TR progresses; at this point, the chemical reactions occurring inside the two sets of batteries differ, resulting in differences in the detected gases.
3. Differences in sensor detection limits and sampling methods. [74] installed 6 sensors approximately 1–2 m above the battery pack for online monitoring. The capture threshold was set such that H_2_ and CO were detected when their concentrations increased by 20 ppm, while for CO_2_, the threshold was 50 ppm. Given that the concentration of each gas varies, the concentration of some gases during TR may be consistently lower than that of others [54,66]. Therefore, the setting of detection limits will affect the capture time of each gas. In contrast, Pan et al. set the sampling point 5 cm above the battery and conducted sampling at 90 s intervals. This may lead to missing the actual generation nodes of some gases. For example, when CO was sampled and detected at 482 s, its concentration had already reached 12 ppm. Although this factor does not objectively change the sequence of gas generation during TR, optimizing the detection limit settings or sampling methods could potentially reduce the time difference in which different gases are successively captured (or detected), even to a negligible extent.

In summary, although there are some divergences regarding the selection of optimal TR gases, a comprehensive analysis of factors such as gas generation sequence, gas concentration, and monitoring feasibility shows that H_2_, CO_2_, CO, electrolyte vapor, and CH_4_ have all undergone careful investigation and experimental verification by researchers. They are considered suitable conventional target gases for TR monitoring, and TR warning experiments in various studies have also confirmed that monitoring for these gases is feasible. There are indeed sequential differences in the generation of different gases; however, in some cases, the impact of this aspect on the efficiency of TR warning does not seem to be as significant as expected. From the perspective of monitoring logic, all existing studies on gas monitoring mention reliance on battery venting. In most cases, after a battery is abused and triggers TR, various gases are continuously generated and gradually accumulate inside the battery through chemical reactions. When the gas volume reaches a certain threshold, the battery’s safety valve opens and venting occurs once or twice (flexible pouch batteries usually can only vent through casing rupture). Currently, the mainstream monitoring method achieves the detection of TR gases precisely by capturing battery vent gas. Since various target gases are mixed in the vent gas, this also ensures the universal effectiveness of monitoring for these gases.

It should be noted, however, that a context-specific analysis remains imperative in practical monitoring scenarios. Factors such as battery type, TR trigger method, battery venting time, and the extent of TR may alter the battery’s venting characteristics (e.g., the concentration of emitted gases, venting time intervals, etc.) [15,54,55,67,71], which in turn leads to differences in the difficulty of monitoring different gases or variations in the final monitoring results. In addition, Batteries can be classified by their packaging forms into prismatic, cylindrical, and pouch (soft-pack) batteries. Due to significant structural differences, they exhibit distinct behaviors during TR, with detailed comparisons as follows [83,84]:

1. Prismatic Batteries

Typically equipped with a pressure relief valve (PRV) that opens when the internal pressure reaches a threshold, allowing controlled gas release through the valve port with a predictable venting path. Minor electrolyte spray may occur, but intense sparking is generally absent. During TR, prismatic batteries undergo minimal casing deformation and generate low expansion force, resulting in relatively controllable hazards and a comparatively longer warning time. Concentrated gas venting enables reliable placement of sensors (e.g., gas or pressure sensors) near the valve port for effective monitoring, making it the most favorable battery structure among the three for implementing safety warning and gas management systems.

**Figure 7:**
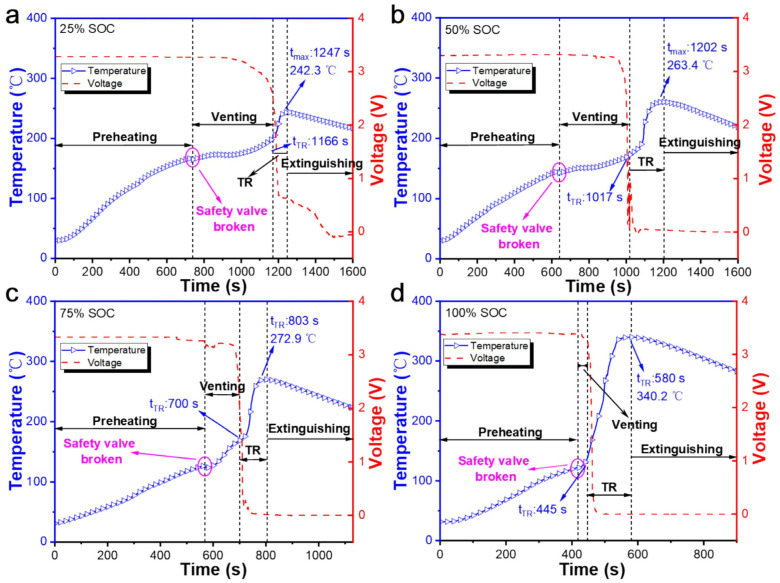
Temperature and voltage curves of a 32 Ah battery with different SOC [85]. (**a**) at 25% SOC; (**b**) 50% SOC; (**c**) 75% SOC; (**d**) 100% SOC. The t_TR_ refers to the moment when dT/dt reaches 1 °C/s.

According to the experimental data of the prismatic LFP battery TR induced by external heating reported by Wang et al. [85], as shown in Figure 7, the higher the SOC, the shorter the time interval from the start of gas emission to TR. Specifically, the difference is approximately 400 s between 25% SOC and 50% SOC, around 150 s at 75% SOC, and only several tens of seconds at 100% SOC. Meanwhile, an increase in SOC advances gas emission and voltage changes, but the arrival time of TR is also correspondingly shortened, resulting in a continuous reduction in the warning window. Thus, the issue of warning time needs to be considered.

2. Cylindrical Batteries

During the TR process, the pressure relief vents at the top of batteries generate an intense axial high-pressure jet. The gas flow typically carries a large number of particles, forming a complex three-dimensional anisotropic flow field. Although the overall rigidity of the steel casing is high enough to resist structural deformation, the rapid jetting process in the late stage of TR can still affect adjacent batteries. Thus, the design of battery packs needs to consider mitigating the thermal shock of the jet axis on adjacent batteries and modules. However, the strong directional characteristic of the jet provides convenience for the directional arrangement of sensors.

**Figure 8:**
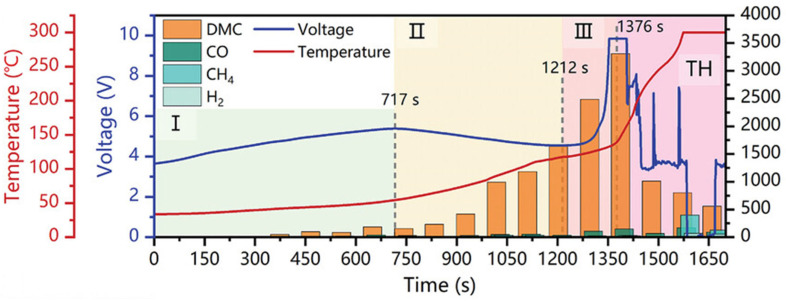
The voltage, surface temperature and gas release condition of the LFP cell during its overcharging process [75]. At 717 s, the LFP voltage reached its first peak; at 1212 s, the LFP voltage began to rise again at an accelerated rate; at 1376 s, the LFP temperature began to increase at an accelerated rate.

For instance, Pan et al. [75] conducted overcharge tests on 18650–type LFP cylindrical batteries. The results in Figure 8 showed that gaseous DMC (390 s), CO (482 s), and CH_4_ (738 s) could be detected at relatively early times; weak white smoke appeared at the battery pressure relief valve at 1212 s. All these phenomena occurred earlier than the actual TR initiation time (self-heating onset time: 1376 s). This study also demonstrated a good warning effect for TR caused by overheating and external short circuits. Therefore, it can be observed that 18650–type LFP cylindrical batteries seem to have a relatively long response window for TR warning.

3. Pouch (Soft-Pack) Batteries

Lacking a fixed pressure relief path, pouch batteries rely entirely on the random rupture of the flexible aluminum-plastic film casing under internal high pressure for gas venting during TR. High-temperature gases and debris are ejected simultaneously, accompanied by obvious sparking. This results in the most significant expansion deformation among the three types, with measured expansion forces far exceeding those of rigid batteries and accompanied by drastic changes in internal resistance. Research by Yu et al. [86] shows that the time it takes for a pouch cell to begin overcharging until TR starts is usually short, only a few dozen seconds. Due to the unpredictable nature of venting location, timing, and direction, gas safety monitoring for pouch batteries poses substantial challenges.

If researchers aim to achieve more efficient and accurate TR warning, they need to conduct specific analyses through targeted experimental design, and flexibly select and design suitable gas sensors based on the gas generation characteristics under different scenarios.

## 5. Overview of Semiconductor Resistive Gas Sensors

Real-time and accurate detection of gases released during the TR process using gas sensors enables battery safety warning [11,45]. Common gas detection technologies include GC, FTIR, GC-MS, Raman spectroscopy, MOS gas sensors, and NDIR technology [8,15]. Considering that a large part of these advanced technical methods are used as tools for studying the gas generation mechanism of TR and for qualitative and quantitative analysis of gases, and some of these detection technologies have high costs, they are difficult to directly apply to the actual monitoring and warning of TR. In contrast, semiconductor gas sensors have advantages such as fast response and recovery speeds, high stability, and relatively low cost, making them an ideal choice for gas detection in the field of TR.

### 5.1. Analysis of the Possibility and Practical Feasibility of Using MOS Gas Sensors to Detect Thermal Runaway in Lithium-Ion Batteries

#### 5.1.1. Analysis of Warning Timeliness Based on Gas Sensing

Several studies have shown that gas sensors targeting characteristic gases (e.g., VOCs, H_2_, CO, CH_4_) can provide a warning window of tens of seconds to 17 min before the initiation of TR [87,88,89,90], which confirms their potential as a TR warning method. In Table 3, the gas production information from seven literatures in different research fields are extracted and summarized, the information in these diverse studies implicitly supports the feasibility of gas monitoring-based methods. However, this warning window is significantly affected by charge rate and heating temperature: the higher the overcharge rate and heating temperature, the earlier the emission and detection of characteristic gases, while the TR initiation time is also correspondingly advanced, ultimately leading to a narrowed warning window. In addition, battery capacity can also affect the TR behavior of the battery [85,91]: large-capacity batteries can significantly extend the TR heating time, delay the onset of both TR and gas emission, and thus obtain a longer warning window.

In summary, the MOS-based gas-sensing method is a highly promising TR warning technology, and its warning timeliness is usually superior to external voltage and temperature measurement methods. However, it should be noted that battery TR characteristics are significantly affected by factors such as charging rate, heating temperature, battery type, and capacity. Signals such as voltage, temperature, and gas will fluctuate accordingly, leading to differences in the duration of the warning window. Therefore, the application scenarios of sensors need further refined research to meet practical requirements.

#### 5.1.2. Discussion on Practical Application Feasibility of MOS-Based Gas Sensors

(1) Performance of the Sensors Themselves

MOS-based gas sensors can achieve rapid and sensitive responses. Studies have shown that their response speed can reach the second level, and the detection limit is as low as the ppb level, which can trigger timely responses after gas emission. Meanwhile, MOS sensors feature miniaturization and low cost, facilitating integration with battery management systems.

(2) Feasibility of Integration and Installation

Extreme temperatures induced by TR are a major concern for sensor applications. However, a gas detection study under overheating conditions confirmed that MOS-based sensors can withstand harsh operating environments [91]. In addition, multiple studies have shown that high-performance sensors can complete alarm responses before the onset of severe temperature rise. Intervention at this time can prevent further deterioration of battery safety conditions, thereby avoiding exposure to high temperatures. Therefore, MOS-based sensors can undertake the monitoring task during the TR process. To further enhance safety, special protective designs can also be implemented to improve the sensor’s applicability. For example, install protective covers with very small apertures to block possible high-temperature particles and flames, extending service life [91].

The installation position of sensors is also crucial. The installation position affects the sensor trigger time, and the sensor closest to the failed cell usually triggers first. However, a research team placed sensors at distances of 1 inch and 9 inches from a battery and found that the response delay was less than 6 s [87]; therefore, as long as the sensors are properly positioned, they can avoid damage during TR without significantly affecting the warning time. In addition, safety monitoring at the battery pack level is also worth attention [13]. Compared with other monitoring methods, gas monitoring typically requires fewer sensors. By optimizing the ventilation within the battery pack and reasonably adjusting the sensor placement, it is possible to accomplish TR warning tasks with fewer sensors.

However, since the operation of MOS materials relies on the adsorption of gas molecules on the sensor material surface and their reactions with active oxygen, they may exhibit cross-response to multiple gases in a complex atmosphere. Such cross-sensitivity in the mixed gas environment of TR is essentially the result of competitive adsorption and synergistic reactions among multi-component gases. It may interfere with the sensor’s selectivity and quantitative detection capability for characteristic gases such as H_2_ and CO, thereby affecting the accuracy and reliability of TR warning. This challenge can be addressed from multiple aspects, including material design and sensing system construction.

### 5.2. Sensing Principle of MOS Gas Sensors

**Figure 9:**
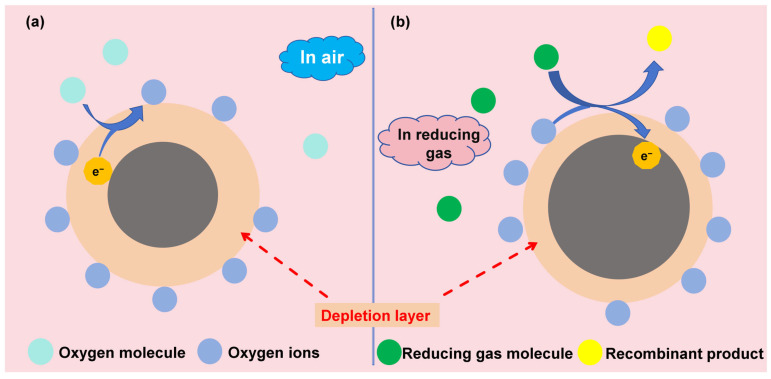
Sensing mechanism of n-type semiconductors for reducing gases (**a**) in air; (**b**) in reducing gas.

The sensing mechanism of semiconductor gas sensors is based on the oxygen adsorption model [93], as demonstrated in Figure 9. In air, oxygen molecules are adsorbed on the surface of the sensor material; by capturing electrons from the conduction band, they form negatively charged oxygen species (e.g., $O_{2}^{-}$, O^−^, or O^2−^). The type of adsorbed oxygen is closely related to the material properties and operating temperature: typically, $O_{2}^{-}$ is dominant from room temperature (RT) to 100 °C, O^−^ is more prevalent between 100 and 300 °C, and O^2−^ becomes the main species above 300 °C [17,94,95].

(17) $$O_{2}\left(\mathrm{gas}\right)\;↔\;O_{2}\left(\mathrm{ads}\right)\;\;\;(T\geq \mathrm{RT})$$

(18) $$O_{2}(\mathrm{ads})+e^{-}\;↔\;O_{2}^{-}\left(\mathrm{ads}\right)\;\;\;(\mathrm{RT}\text{-}100\;{}^{\circ}C)$$

(19) $$O_{2}(\mathrm{ads})+2e^{-}\;↔\;2O^{-}\left(\mathrm{ads}\right)\;\;\;(100–300\;{}^{\circ}C)$$

(20) $$O^{-}\;(\mathrm{ads})+e^{-}\;↔\;O^{2-}\left(\mathrm{ads}\right)\;\;\;(T>300\;{}^{\circ}C)$$

Semiconductor materials can be divided into two categories based on their charge carriers. The first is n-type semiconductors, which are dominated by electron carriers in their base state (e.g., TiO_2_, SnO_2_ [96], In_2_O_3_, ZnO [97,98], Fe_2_O_3_). When O_2_ is adsorbed on the surface of an n-type semiconductor material, electrons are captured by O_2_, resulting in the formation of an electron depletion layer (EDL) with a low electron concentration on the semiconductor surface. The other is p-type semiconductors, where hole carriers are predominant in the base state (e.g., CoO, NiO). When oxygen is adsorbed on the surface and forms oxygen species, O_2_ captures electrons; after electrons are removed, a hole accumulation layer (HAL) is formed on the material surface [99]. In practical gas detection scenarios, target gas molecules are adsorbed onto the surface of semiconductor materials and undergo redox reactions with the surface-active oxygen species. This reaction releases the electrons previously captured by the oxygen species back into the semiconductor’s conduction band, causing the surface EDL to shrink. Meanwhile, oxygen vacancies are reformed on the material surface, the Fermi level and conduction band return to their initial states, and the resistivity is eventually restored. All these changes alter the conductivity of the MOS material [100]. Based on the aforementioned sensing principle, by real-time measurement of the variation patterns in the resistance or conductivity of the semiconductor material, a quantitative correlation between the resistance (or conductivity) and the target gas concentration can be established. This, in turn, enables the qualitative identification and quantitative detection of the target gas.

### 5.3. Performance Evaluation Metrics for MOS Gas Sensors

The commonly used performance evaluation criteria for gas sensors are presented as follows:

(1) Response: When a sensor is exposed to the target gas, it undergoes changes in resistance or current. The magnitude of the response value reflects the amplitude of these signal variations. There are two methods to calculate the response value:

(I) Ratio of the steady-state resistance of the material in air (R_a_) to its resistance when exposed to the target gas (R_g_).

For n-type semiconductor sensing materials, when the test gas is a reducing gas, the response value is calculated using the formula:

Response = R_g_/R_a_ (21)

When exposed to an oxidizing gas, the formula is:

Response = R_a_/R_g_ (22)

For p-type semiconductor sensing materials, the calculation formulas are the reverse of those for n-type semiconductors.

(II) Relative Response:

(23) Response =Ra−RgRa×100%   (Reducing gases)

(24) Response=Rg−RaRa×100%   (Oxidizing gases)

(2) Response Time and Recovery Time

Response time refers to the duration required for the resistance of the material to reach 90% of its final equilibrium value R_g_ from its steady-state resistance in air R_a_, starting from the moment the material comes into contact with the target gas. Recovery time refers to the duration required for the resistance of the sensing material to recover by 90% from R_g_ back to the steady-state resistance in air R_a_ after the target gas is removed.

(3) Selectivity: Selectivity refers to the capability of a sensor to respond to the target gas or resist interference in a mixed gas environment. It is typically evaluated by comparing the sensor’s response to the target gas with its response to other gases at the same concentration.
(4) Stability. Stability is a critical parameter for the industrialization and commercialization of sensing materials. It can be divided into two categories:

Repeatability: Refers to the stability of the sensing material during operation over a specific period.

Long-term stability: Refers to the ability of the sensing material to maintain its gas-sensing performance after prolonged service in the environment.

(5) Limit of Detection (LOD): LOD refers to the minimum concentration of the target gas that can be detected by the sensor. A lower LOD value indicates higher sensitivity of the sensor.
(6) Optimal Operating Temperature

Optimal operating temperature refers to the temperature corresponding to the maximum response value at a given gas concentration. The sensing characteristics of MOS materials depend on the carrier concentration, which is related to the operating temperature. If the temperature is too low, the activity of the sensing material cannot be fully activated. If the temperature is too high, the gas adsorption capacity will be weakened, which is unfavorable for the response.

## 6. Research Progress on Gas-Sensing Materials for TR Warning

Currently, to achieve effective monitoring and warning of TR, a subset of studies has focused on the sensing of characteristic gases emitted during TR. The core approach involves enhancing the sensing performance of gas-sensing materials through various modification strategies. Specific modification methods include regulating preparation processes or experimental methods to optimize material morphology, doping single metals or bimetals to adjust the electronic structure of materials, and constructing composite material systems to form heterojunctions. Below, the research progress of gas-sensing materials in the field of TR warning will be elaborated in detail, targeting five key characteristic gases (H_2_, electrolyte vapor, CO, CO_2_ and CH_4_) released during the TR process.

### 6.1. Research Progress of Gas-Sensitive Materials for H_2_

In the field of H_2_ detection during LIB TR, research on H_2_-sensing materials primarily focuses on optimization strategies such as metal doping, heterojunction construction, and composite material design. For example, MOS materials (e.g., ZnO, TiO_2_) are modified via single-metal or bimetal doping or modification, which effectively enhances the materials’ response capability to H_2_ or reduces the operating temperature of the sensing materials. Heterojunctions are constructed through material compounding to overcome the limitations of pure components; a typical case is the combination of In_2_O_3_ and NiO, which addresses the issue of poor responsiveness in pure In_2_O_3_ or NiO. Additionally, some studies have explored the composite application of H_2_-sensing materials with emerging functional materials, where materials like MXene and g-C_3_N_4_ are combined with SnO_2_. Each type of modified material exhibits unique advantages, providing solutions for H_2_ detection during TR.

**Figure 10:**
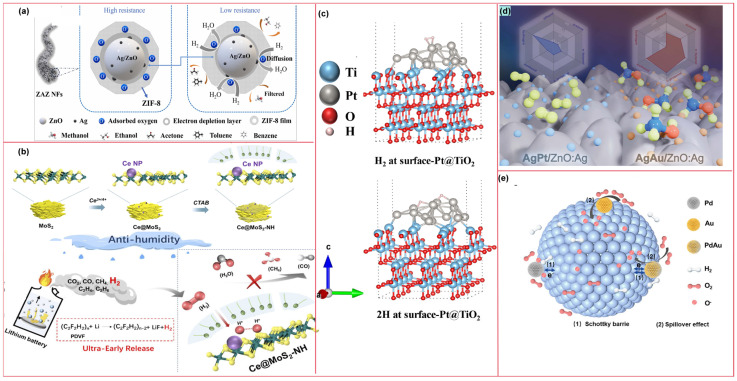
(**a**) Schematic illustration of the gas-sensing mechanism for ZAZ NF [101]; (**b**) The self-assembly process of hydrophobic surfactant Ce@MoS_2_-NH and H_2_ monitoring performance against humidity and interference [102]; (**c**) The adsorption and splitting of H_2_ atoms on Pt/TiO_2_ [103]; (**d**) ZnO: Ag materials modified with AgAu and AgPt bimetallic nanoparticles [104]; (**e**) Gas-sensing mechanism of PdAu-In_2_O_3_ [105].

The metal doping strategy is typically effective in enhancing gas-sensing performance. In 2024, Wang et al. [101], taking H_2_ as the target monitoring gas, designed ZIF-8 supported Ag/ZnO electrospun nanofibers (ZAZ NFs) for battery safety warning. This composite material features a core–shell structure, as illustrated in Figure 10a. Benefiting from the gas enrichment and sieving effect of the ZIF-8 shell, the catalytic sensitization effect of noble metal Ag, and the abundant active sites provided by both components, the material enables high-performance detection of H_2_ at low concentrations. The sensor achieved a ppb-level limit of detection for H_2_ in test, along with an extremely fast response time of 9 s and excellent moisture resistance. The authors also conducted a TR safety warning simulation using pouch LIB. The results showed that the sensor could issue a warning 67.79 s before battery bulging occurred, demonstrating favorable safety monitoring capability. To achieve warning of LIB TR. Hou et al. [102] also adopted the metal doping strategy and developed a H_2_ sensor that operates at a relatively low temperature (100 °C) and exhibits excellent moisture resistance. This sensor uses Ce-doped MoS_2_ as the gas-sensing material; Ce doping optimizes the electronic structure of MoS_2_, thereby enhancing its sensing performance. The sensor demonstrates high selectivity and a fast response (11 s) toward H_2_. Moreover, as shown in Figure 10b, they incorporated CTAB into the material to form a hydrophobic layer. This layer enhances the hydrophobicity of the gas-sensing material, endowing it with moisture resistance. In TR experiments triggered by overcharging or overheating of LIBs, the H_2_ sensor issued a warning 76 s earlier than traditional temperature sensors, indicating favorable warning capability. However, the long-term stability of this sensor needs improvement, and it has the drawback of high-temperature sensitivity (100 ppm H_2_/°C). Temperature fluctuations easily lead to detection deviations. In the future, material modification or process optimization may be required to further enhance its temperature stability.

Zhang et al. [103] employed both metal doping and modification strategies in their research. They developed an MEMS H_2_ sensor capable of operating at room temperature (40 °C), using Pt-modified Nb-doped TiO_2_ nanosheets as the sensing material. As illustrated in Figure 10c, Nb doping introduced a large number of oxygen vacancies, which improved the sensor’s response performance; meanwhile, surface modification with Pt nanoparticles successfully reduced the operating temperature and further increased the response value. In contrast, Vahl et al. [104] adopted a bimetallic modification strategy to optimize the gas-sensing performance of single-metal-doped sensitive materials. They used Ag Au and AgPt bimetallic nanoparticles to modify Ag-doped ZnO (ZnO:Ag) columnar thin films, respectively. As depicted in Figure 10d, this modification not only enhanced the sensing performance of ZnO:Ag sensitive materials but also altered their gas selectivity: compared with the original ZnO:Ag thin films, AgAu modification increased the sensor’s response to 100 ppm of VOCs (such as ethanol and acetone) by 2.8 to 6 times; AgPt modification, however, significantly reduced the response to VOCs while markedly improving the response to H_2_. This phenomenon is attributed to the fact that different dopants alter the gas adsorption properties of the material. Based on this, the research team concluded that the selectivity of ZnO:Ag thin films can be customized via bimetallic nanoparticle modification, enabling the detection of H_2_ released during TR. Li et al. [105] reported a study on H_2_ detection that combines a temperature-modulated PdAu-In_2_O_3_ sensor array with machine learning algorithms. Leveraging the spillover effect and sensitization of Au and Pd nanoparticles, the optimized Pd_2_Au_1_-In_2_O_3_ material exhibits excellent gas-sensing performance: at a relatively low temperature of 130 °C, its response to 100 ppm H_2_ reaches 24.61, with a detection limit as low as 100 ppb. The gas-sensing mechanism of this material is illustrated in Figure 10e. They also constructed a sensor array by adjusting the modification ratio and combined it with a feature recognition algorithm, which enabled the classification of H_2_, ethanol, and their mixtures, as well as the prediction of their concentrations. Furthermore, they developed an electronic nose system for real-time monitoring of H_2_. This research holds significant implications for the realization of battery safety detection and artificial intelligence-driven gas-sensing technologies.

**Figure 11:**
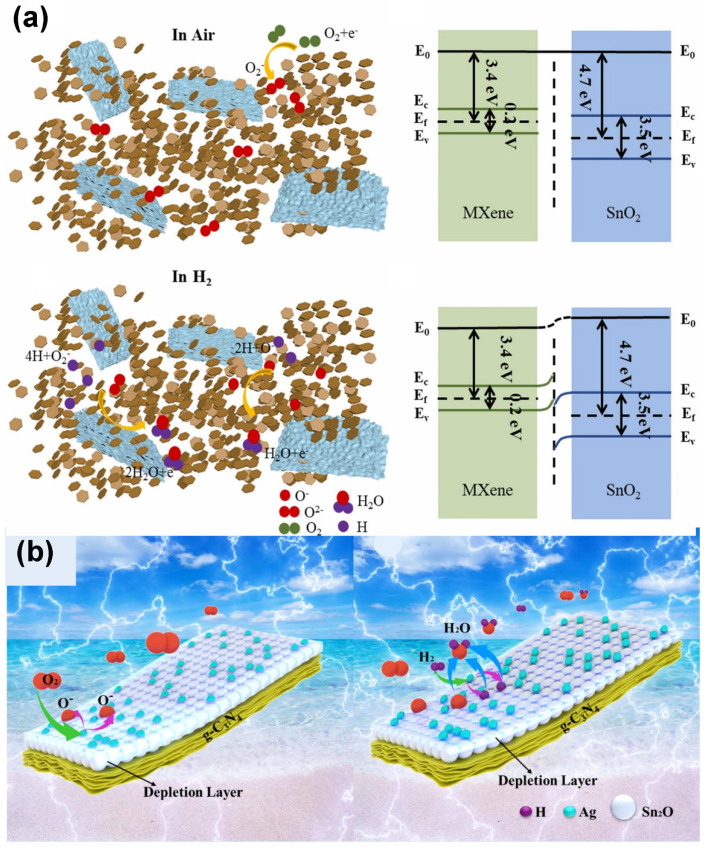
(**a**) The H_2_ sensing mechanism of MXene-SnO_2_ nanocomposite materials [107]; (**b**) Illustration of H_2_ sensing mechanism for CSE gas sensor [108].

Another effective approach to improve sensing performance is constructing heterojunctions using composite materials. Jiang et al. [106] adopted a heterojunction construction strategy to fabricate a H_2_ sensor based on In_2_O_3_/NiO nanosphere composites. This sensor operates at room temperature and outperforms pure In_2_O_3_ and pure NiO samples, with significantly shortened response time and recovery time. Studies revealed that the formation of heterojunctions facilitates charge transfer: strong electronic interactions between In and Ni atoms enhance catalyst activity, while the spherical agglomerated structure provides abundant active sites. These factors collectively improve the gas-sensing properties of the sensor. Chen et al. [107] synthesized Ti_3_C_2_Tx MXene-SnO_2_ nanocomposite thin films via a hydrothermal method, combining a MXene material with SnO_2_ hexagonal nanosheets (Figure 11a). Benefiting from the high specific surface area of MXene and the formation of heterojunctions in the composite, the material exhibits significantly enhanced H_2_ sensing performance. The sensor exhibits advantages including fast response/recovery speeds, higher selectivity, and better stability, with a theoretical detection limit of 1.81 ppm.

Shao et al. [108] combined graphitic carbon nitride (g-C_3_N_4_) with SnO_2_ semiconductor material and incorporated noble metal Ag to develop a composite material with a novel intercalated structure. This sensitive material was prepared via layer-by-layer spin coating, forming a sandwich structure consisting of a catalytic sensitization layer (Ag nanoparticles), a gas-sensitive layer (SnO_2_), and an electron-donating layer (g-C_3_N_4_). The structure and sensing mechanism are illustrated in Figure 11b. At 300 °C, the Ag nanoparticles are in an amorphous state, exhibiting more defect sites and higher reactivity. The synergistic enhancement effect generated by the three distinct functional layers collectively endows the sensor with excellent sensing performance: it shows high selectivity and sensitivity toward H_2_, with extremely short response/recovery times (3 s/4 s) and a detection limit as low as 30 ppb. This provides a new approach for the effective monitoring of TR in LIBs.

Overall, research on H_2_ sensing has focused primarily on the doping and modification of rare metals and noble metals, which is an extremely effective strategy that can significantly enhance the response capability of sensing materials and reduce the sensor’s operating temperature to a certain extent. For instance, the operating temperatures of the aforementioned rare metal-doped and noble metal-doped materials (e.g., Nb, Pd, Ag, Pt, Au) range from 100 to 260 °C, mostly lower than those required for composite materials. This is attributed to the electronic modulation effect of noble metals on the materials and their catalytic sensitization towards H_2_. Furthermore, applying metal doping on the basis of morphology regulation may further improve their gas-sensing performance. Heterojunction construction and material composite strategies are also highly effective modification approaches for H_2_ sensing, primarily by improving the charge transfer performance of materials to enhance gas-sensing properties. However, compared with studies adopting metal doping strategies, a simple composite of metal oxide materials is rarely able to achieve superior gas-sensing performance. Nevertheless, appropriate morphological structure design, such as creating nanostructures (e.g., nanospheres, nanofibers, nanorods, nanoparticles), regulating the exposure of active sites and improving the transport of gas molecules, may enhance the gas-sensing performance of composite materials.

An increasing number of studies are now integrating different strategies, such as combining morphology regulation, noble metal doping, functionalized materials, and heterojunction construction, to develop high-performance H_2_ sensors.

### 6.2. Research Progress of Gas-Sensitive Materials for Electrolyte Vapor

The research progress of semiconductor sensors for five typical electrolyte vapors (i.e., DMC, DEC, EMC, DME, and DME) is discussed in the following sections.

#### 6.2.1. Research Progress of Gas-Sensitive Materials for DMC Vapor

As summarized in Table 4, currently reported DMC gas-sensitive materials cover oxides (e.g., Bi_2_O_3_, SnO_2_), chlorides (Cs_2_SnCl_6_), and ion-modified MOFs. Modification strategies focus on doping or surface modification with noble metals (e.g., Ag) and transition metals; by regulating the electronic structure of materials, the number of active sites, and gas adsorption kinetics, these strategies enhance the sensitivity, selectivity, and response speed toward DMC.

**Figure 12:**
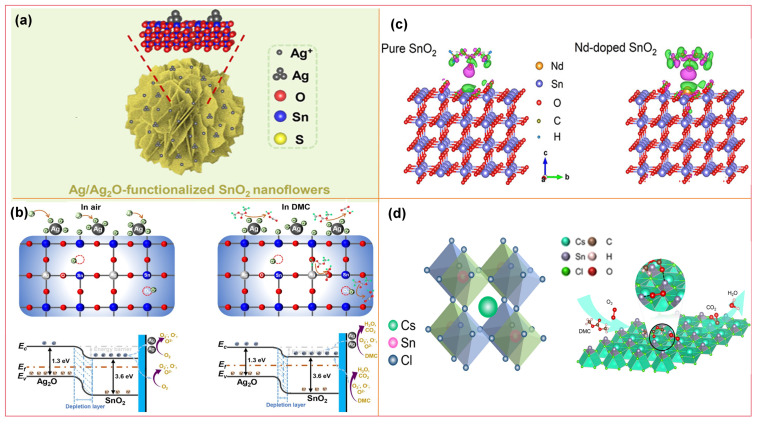
(**a**) Ag@Ag_2_O Functionalized SnO_2_ Nanoflower [109]; (**b**) The H_2_ sensing mechanism of SnO_2_ nanocomposite materials [109]; (**c**) Nd-doped SnO_2_ materials enhanced DMC adsorption performance [110]; (**d**) The structure of Cs_2_SnCl_6_ material and the gas sensitivity mechanism of DMC [79].

In 2020, Lu et al. [112] proposed developing gas sensors for detecting electrolyte leakage to address LIB safety issues. They fabricated ion-conductive metal–organic framework (IC-MOF) nanofilms based on Cu-TCPP via a modified liquid-interface spray method. The constructed sensor exhibits excellent sensing performance: it can rapidly and effectively detect ultra-low concentrations of DMC (50 ppb), with long-term stability (6 months). In experiments simulating electrolyte leakage, real-time detection was achieved, and its warning time was significantly earlier than that of abnormal voltage signals. The outstanding sensing performance of the sensor can be attributed to the Cu ions present in the MOF. Pan et al. [75] synthesized Bi_2_O_3_ nanosheets via a one-step solvothermal method and fabricated a semiconductor sensor for DMC detection. Results show that the sensor exhibits high selectivity toward DMC, along with high sensitivity and fast response capability. Its detection limit is as low as 50 ppb, making it suitable for trace DMC monitoring. In simulation tests of TR triggered by overcharging or overheating, the sensor demonstrates excellent warning performance, issuing a TR warning 15 min in advance. The excellent DMC selectivity stems from the catalytic decomposition of partial DMC molecules on the Bi_2_O_3_ surface, and the resulting intermediate (methyl monocarbonate, MMC) contributes to the enhanced response. Sun et al. [109] introduced noble metal Ag doping and synthesized Ag@Ag_2_O functionalized SnO_2_ nanoflower-like composites. As illustrated in Figure 12a, Ag aggregates into larger nanoparticles on the surface of SnO_2_ nanoflowers. The sensor shows a sensitive response to different electrolyte vapors in LIB, with an ultra-high response value of 106 toward DMC (100 ppm), a response/recovery time of only 28/55 s, and a detection limit as low as 11.76 ppb. In simulated electrolyte leakage experiments, the sensor enables highly sensitive and rapid response to trace electrolytes.

Wan et al. conducted two studies on metal-doped modification of SnO_2_ materials for DMC gas sensing. In both studies, the sensors exhibited a relatively low operating temperature (150 °C). In their initial study [110], a type of rare-earth neodymium-doped SnO_2_ hollow nanofiber material was reported. Nd doping endowed the material with a porous hollow structure and more oxygen vacancies. It also significantly improved the adsorption capacity of SnO_2_ for DMC, as illustrated in Figure 12c. The sensor’s detection limit was as low as 20 ppb, which enables the detection of trace gases. However, the Nd-SnO_2_ material was found to have relatively long response and recovery times for DMC. Thus, in another study [111], they explored a new bimetallic doping modification method and synthesized a Co/Pd-doped SnO_2_ nanocomposite. This synthesis successfully shortened the response time. The sensor based on this material exhibited good DMC leakage monitoring performance. It could respond to DMC at a concentration as low as 500 ppb. This is attributed to the increase in active sites and the sensitization effect of noble metals. When detecting electrolyte leakage in an actual LIB, the sensor could make a timely response within 50 s after leakage.

Zhou et al. [79] synthesized a lead-free metal halide perovskite (MHP) material with an octahedral structure Cs_2_SnCl_6_, as illustrated in Figure 12d. The gas sensor exhibited good adsorption performance toward DMC molecules. It showed good selectivity, with a response value of 7.05 toward 100 ppm DMC, and a response/recovery time of 82 s/83 s toward 20 ppm DMC. In addition, they proposed a new insight into the adsorption and decomposition mechanisms of MHP-based gas sensing. This provides guidance for the design of high-performance lead-free MHP gas-sensing materials.

#### 6.2.2. Research Progress of Gas-Sensitive Materials for DME Vapor

Research on DME gas-sensitive materials is limited (Table 5). These studies mainly use the strategy of constructing heterojunctions with metal oxide composites to enhance the gas sensitivity of semiconductor materials.

**Figure 13:**
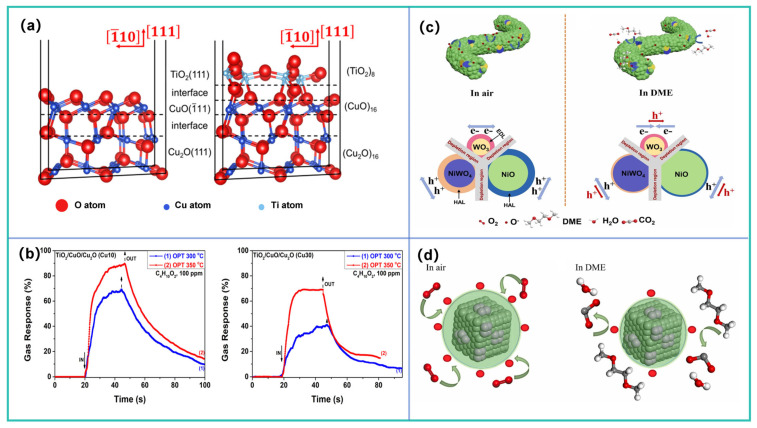
(**a**) Schematic diagram of the ternary TiO_2_ (111)/CuO ($\bar{11}1$)/Cu_2_O (111) composite material and (**b**) the fast response characteristics of the composite material [113]; (**c**) The DME sensing mechanism of NiO/Si-NiWO_4_/WO_3_ NFs [114]; (**d**) The gas-sensing mechanism of CuSnO_3_ modified In_2_O_3_ [115].

Lupan et al. [113] developed a sensor based on ternary TiO_2_ (111)/CuO ($\bar{11}1$)/Cu_2_O (111) heterojunction thin film materials via magnetron sputtering, as illustrated in Figure 13a,b. The formation of heterojunctions improved adsorption properties: it enhanced the material’s adsorption toward DME while inhibiting water adsorption, thus endowing the sensor with good DME selectivity and moisture resistance. The sensor had a relatively fast response time of 11.1 s, and could produce an effective response at a low concentration of 1 ppm. Gao et al. [114] proposed a sensing strategy for constructing heterojunction composites based on defect-rich polyoxometalates (POMs). They prepared ternary NiO/Si−NiWO_4_/WO_3_ heterojunction nanofibers for DME sensing. The composite had a high specific surface area, as shown in Figure 13c. The p-p-n ternary heterojunction enhanced oxygen adsorption and carrier transfer capabilities, which significantly improved sensing performance. For 25 ppm DME, the sensor had a response/recovery time of 22 s/86 s and a detection limit as low as 300 ppb. As illustrated in Figure 13d, Zhu et al. [115] designed a cubic In_2_O_3_ composite modified with amorphous bimetallic oxide CuSnO_3_ (CSO). The composite had a response value of 6.2 toward 20 ppm DME. Studies revealed that CSO increased the number of defect sites and oxygen vacancy concentration; combined with the electronic modulation effect of the amorphous heterojunction and the bimetallic synergistic catalysis of CSO, these factors collectively enhanced the material’s response capability. Overall, CSO/In_2_O_3_ not only had a low detection limit (0.1 ppm) and a low operating temperature (220 °C), but also exhibited fast response capability (with response and recovery times of 19 s and 9 s, respectively). It also had a certain tolerance to humidity interference, indicating broad application potential.

**Table 5:** Summary of the performance of DME sensing materials.

Material	Target Gas	Conc. (ppm)	Response	T (°C)	Res/Rec. (s)	LOD (ppb)	Ref.
TiO_2_/CuO/Cu_2_O (Cu_10_)	DME	100	89%	350	11.1/56	1 ppm	[113]
NiO/Si-NiWO_4_/WO_3_-3	DME	100	53.0	300	22/86 (25 ppm)	300	[114]
CSO/In_2_O_3_-2	DME	20	6.2	220	19/9 (10 ppm)	100	[115]

#### 6.2.3. Research Progress of Gas-Sensitive Materials for EMC Vapor

In gas-sensing research on EMC vapor, researchers mostly use metal oxides as sensing materials. Besides metal doping modification and composite material construction for heterojunctions, some studies adopt the sacrificial template method to develop SnO_2_ nanomaterials with controlled structures, aiming to achieve high-performance EMC sensing. A summary of EMC gas sensors (including a CsPbBr_3_@In sensor mentioned later) is provided in Table 6.

Liu et al. [116] adopted a metal doping modification strategy and developed a Pd-doped WO_3_ hollow microsphere material for detecting EMC gas in electrolytes. Pd doping enhanced the gas-sensing performance of the material. The gas sensor exhibited good gas selectivity toward EMC: its response value toward 10 ppm EMC reached as high as 17.8, and after Pd doping, its response time was shortened from 45 s to 19 s. It still maintained a 45% response toward EMC at a concentration as low as 100 ppb. The excellent sensing performance of the sensor can be attributed to the large specific surface area of the hollow microspheres, as well as the catalytic and electronic sensitization effects of Pd.

Besides metal doping modification, some studies have focused on preparing SnO_2_ nanomaterials via template methods to develop high-performance semiconductor sensors. With the spatial confinement and structure-directing effects of sacrificial templates, nanomaterials with controlled morphology, porosity, and surface area can be fabricated, realizing the optimization of gas-sensing performance.

**Figure 14:**
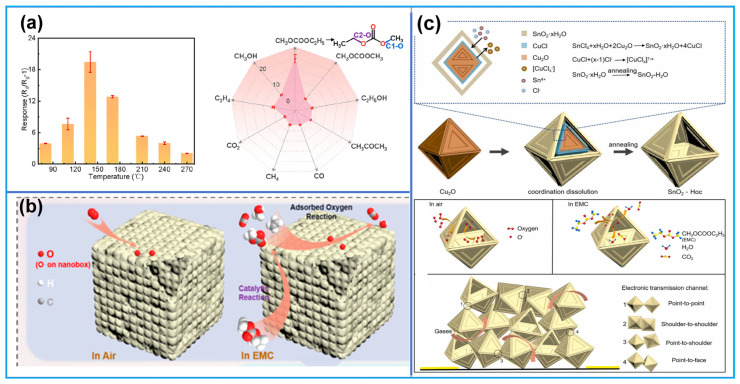
(**a**) EMC-sensing performance of SnO_2_ nanoboxes [117]; (**b**) Gas-sensing mechanism. (**c**) SnO_2_ nanobox-based sensor [117]; (**c**) Schematic illustration of the formation of SnO_2_-Hoc by coordination etching and gas-sensing mechanism [118].

In 2024, Su et al. [117] used cubic Cu_2_O as a template and prepared 3D hollow SnO_2_ nanoboxes via a coordination dissolution method, as illustrated in Figure 14a,b. The material exhibited a large specific surface area (54.5 m^2^/g) and a small grain size (5.2 nm). At a relatively low temperature of 140 °C, the sensor showed a response value as high as 32.46 toward 20 ppm EMC, along with an ultra-low detection limit of 10 ppb and excellent selectivity. The high selectivity is attributed to the favorable reducibility of EMC itself and the response-enhancing effect of intermediates generated from EMC’s catalytic decomposition, while the outstanding sensing performance is attributed to the active sites provided by the large specific surface area and high size/thickness ratio of the SnO_2_ nanoboxes. The sensor could respond rapidly to leakage signals. In another study published by Su et al.in the same year [118], they adopted the template-based preparation strategy: a concave octahedral Cu_2_O template was synthesized, and uniform-sized concave octahedral hollow SnO_2_ nanocages were prepared via coordination etching, as shown in Figure 14c. The hollow concave octahedral structure had a large specific surface area and porous structure, which promoted the diffusion of EMC. Moreover, the energy barrier for the surface reaction of EMC was relatively low, endowing the sensor with good selectivity toward EMC. The sensor had a response value of 7.24 toward 10 ppm EMC and a theoretical detection limit as low as 160 ppb. However, it had a drawback of relatively long response and recovery times (100 s/930 s), which could potentially be improved through certain modification methods.

**Figure 15:**
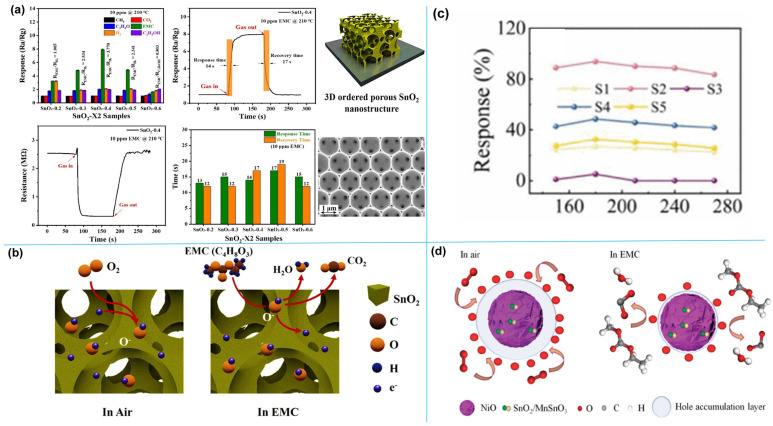
(**a**) The sensing performance and morphology of SnO_2_ material [119]; (**b**) The gas-sensing mechanism for EMC [119]; (**c**) The temperature drift resistance characteristics of MnSnO_3-x_/NiO and (**d**) the gas-sensing mechanism [120].

In 2025, Cao et al. [119] synthesized 3D ordered porous SnO_2_ nanomaterials via a polystyrene sphere template self-assembly method, as illustrated in Figure 15a. They systematically investigated the effects of template size on gas-sensing performance and determined the optimal preparation conditions. Results showed that due to the high specific surface area and ordered mesoporous network structure of the nanomaterial, the SnO_2_ gas sensor exhibited extremely fast response/recovery times (14 s/17 s) and a detection limit as low as 500 ppb. However, the 3D structure of this material is significantly affected by changes in humidity.

Developing sensing materials with both excellent stability and sensitivity is also a research direction of great concern. Due to the influence of heating devices or changes in ambient temperature, LIB TR gas sensors may have baseline thermal drift issues. This affects the stability and sensitivity of the sensors. Currently, drift compensation algorithms and heating electrode optimization technologies are usually used to address this challenge, but these methods are relatively complex to implement.

To solve this problem, Zhu et al. [120] proposed an EMC gas sensor that overcomes baseline thermal drift, achieving a good combination of high sensitivity and high stability advantages. In this study, a strategy of constructing heterojunctions by modifying NiO with amorphous bimetallic oxide MnSnO_3−x_ was adopted, and uniformly dispersed MnSnO_3−x_/NiO nanoflower-like gas-sensitive materials were synthesized via a hydrothermal method. At a relatively low operating temperature (180 °C), the sensor exhibited excellent selectivity toward EMC. The formation of amorphous–crystalline heterojunctions shortened the response time to 34 s, with a detection limit as low as 0.2 ppm. Moreover, as illustrated in Figure 15c, the sensor showed a good suppression effect on drift caused by temperature disturbance. Within a wide temperature range of 150–270 °C, the standard deviation of the response value toward 10 ppm EMC gas was only 3.7%. Studies show that the improved sensitivity is attributed to the heterojunctions formed by the introduction of amorphous MnSnO_3_ and crystalline SnO_2_, which change the band structure; the excellent suppression of sensing thermal drift is attributed to the grain boundary barrier and electronic properties of the unique flower-like layered NiO.

#### 6.2.4. Research Progress of Gas-Sensitive Materials for DEC Vapor

DEC is also a common component in electrolyte solvents, but research on DEC is relatively limited. In 2022, Li et al. [122] synthesized CeO_2_-loaded In_2_O_3_ hollow spheres via a hydrothermal method for the detection of DEC. Studies showed that the incorporation of CeO_2_ significantly improved the sensing performance: at an operating temperature of 200 °C, the sensor had a response value of 5.2 toward 100 ppm DEC, along with extremely fast response and recovery times (2 s/20 s) and a detection limit as low as 100 ppb. These results confirm that it possesses excellent gas-sensing performance. Although this study lacks research on gas selectivity and an explanation for the mechanism behind the enhanced gas-sensing performance, its good performance indicates that the sensor has application potential in TR monitoring.

Overall, for the detection of electrolyte vapor, material design focuses more on the construction of composite heterojunctions and the combination of material morphology regulation strategies. Specifically, research on gas-sensing materials for DMC often adopts the method of combining metal-modified metal oxide materials with morphology regulation, which has achieved remarkable results. For instance, Nd-SnO_2_ and Co/Pd-SnO_2_ developed by Wan et al. can exhibit good responses at a low operating temperature (150 °C) and low concentration (1 ppm). Among them, Nd-SnO_2_ possesses long-term stability of 10 cycles, which is crucial for its practical application in the field of battery safety. The Ag@Ag_2_O-SnO_2_ nanoflower material developed by Sun et al. features a low detection limit (11.76 ppb), along with rapid response (28 s) and high response value (106). However, when considering all performance indicators, the optimal performance comes from the study of Bi_2_O_3_ nanosheets by Pan et al. This material is simply prepared, exhibits excellent comprehensive performance, including good responsiveness and rapid response capability. Therefore, future research may consider optimizing the selection of MOS materials, and incorporating morphology regulation and metal doping on this basis, which is expected to yield favorable results.

The vast majority of EMC sensors can detect at low concentrations (10 ppm) with good responsiveness and fast response speed. This means they can respond when the battery exhibits slight venting, which is attributed to the strong reducibility of EMC and the low energy barrier for surface reactions. However, these sensors suffer from the problem of long recovery time. Notably, the study by Cao et al. [119], which prepared SnO_2_ materials via a polystyrene sphere template self-assembly method, seems to have addressed this issue (response time/recovery time: 14 s/17 s), and this approach is worthy of further reference. Regarding DME and DEC, there are relatively few studies on gas-sensing materials to date. Nevertheless, all existing studies focus on material composition, and even two studies have adopted the ternary heterojunction construction strategy. These modification methods have achieved promising results: for example, response time, a key indicator for TR monitoring, is generally fast (11.1–22 s), which is conducive to safety warning. However, the operating temperatures of these materials are relatively high (220–350 °C), and further exploration and optimization are required in the future.

It should be noted that for the monitoring of electrolyte vapor, attention should be paid to the fact that different batteries may have varying electrolyte contents during packaging. In addition, their boiling points also differ, which leads to differences in emission concentrations. Therefore, it is inappropriate to generalize all indicators uniformly, and flexible setting of detection limits is necessary, which requires further investigation through experiments.

### 6.3. Research Progress of Gas-Sensitive Materials for CH_4_

**Figure 16:**
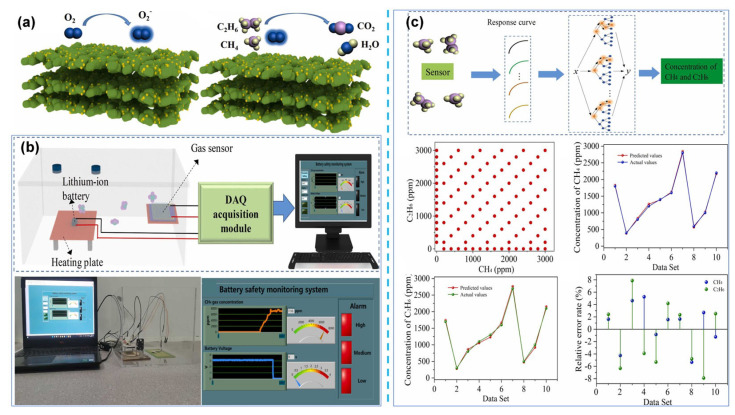
(**a**) Gas-sensing mechanism of V_2_O_5_/NiO materials [124]; (**b**) Schematic diagram of the sensor, TR simulation monitoring experiments [124]; (**c**) Machine learning algorithm and concentration prediction model [125].

Methane is also a typical gas generated during the TR process. Kim et al. [123] have previously reported a V_2_CT_x_ MXene gas sensor, which exhibits extremely high responsiveness to non-polar gases (including hydrogen and methane). Wang et al. [124] designed a methane sensor based on V_2_O_5_/NiO nanocomposites using V_2_CT_x_ MXene and MOFs as raw materials. Its gas-sensing mechanism is illustrated in Figure 16a. The formation of p-n heterojunctions significantly improved the sensing performance toward CH_4_. At an operating temperature of 200 °C, the sensor had a response value of 57% toward 4000 ppm CH_4_, along with good selectivity and long-term stability. However, the sensor also showed good responsiveness to C_2_H_6_ at 350 °C. To address the issue of gas cross-sensitivity, the team combined a single sensor with a random forest (RF) machine learning algorithm and constructed a mixed gas concentration prediction model, as shown in Figure 16c. The average relative error of this model was less than 4.8%, enabling accurate detection in complex gas environments. In LIB TR simulation experiments, the sensor successfully detected the CH_4_ signal released during the TR process, which was significantly earlier than the voltage signal. This verified its feasibility in early fault warning of batteries.

Liu et al. [125] developed a sensor based on SnO_2_/Zn_2_SnO_4_ nanocomposites. This sensor exhibited excellent methane sensing performance: it showed ultra-fast response/recovery times (1 s/9 s) toward 500 ppm CH_4_, with a response value of 4.16, which was significantly better than that of pure SnO_2_ and pure Zn_2_SnO_4_ sensors. It also had a low detection limit of 5 ppm. Its good sensing performance indicates that the sensor has potential application value in warning of CH_4_ gas during the TR process.

### 6.4. Research Progress of Gas-Sensitive Materials for CO and CO_2_

Carbon monoxide (CO) and carbon dioxide (CO_2_) are also two common gases generated during the TR of batteries. They are mainly released during the processes of SEI decomposition, electrolyte solvent decomposition, oxidation of electrode materials, and combustion of flammable gases. During TR, the release ratio of CO to CO_2_ is closely related to the oxygen (O_2_) content in the battery and the intense oxidation process during the TR process. However, few studies have been reported on the application of CO_2_ or CO gas sensors in the monitoring and warning of TR, and the application of semiconductor-based sensors in this field is almost non-existent. Considering that research on semiconductor gas sensors in non-TR fields is already highly mature, these sensors hold potential for TR monitoring, with further optimization, they can be applied to practical TR monitoring. Therefore, in the following sections, we will not only introduce the research progress of CO and CO_2_ sensors in TR monitoring but also present studies that exhibit potential for application in this area.

First, we focus on studies related to CO_2_-based warning for TR. As one of the main components of released gases, CO_2_ can be accurately monitored with low-cost sensors. Currently, the commonly used CO_2_ sensors include electrochemical sensors, semiconductor sensors, non-dispersive infrared (NDIR) sensors, and optical fiber sensors [15]. The existing warning studies targeting CO_2_ mainly include the following: the detection method for LIB TR based on a NDIR CO_2_ sensor proposed by Cai et al. [76] in 2021; and the NDIR gas-sensing system composed of pyroelectric infrared detectors proposed by Han et al. [126] in 2023, which is used to monitor CO_2_ and CH_4_ and provide warning for TR.

In addition, to achieve CO_2_ gas sensing, Choudhary et al. [127] prepared yttrium-doped ZnO:CdO (YZC) nanocomposite thin films via a sol–gel method, which exhibited a cauliflower-like morphology. This study achieved CO_2_ gas sensing at room temperature (27 °C): the maximum response value of the sensor toward 500 ppm CO_2_ was 9, with fast response and recovery times of 4 s and 2 s, respectively. Haldar et al. [128] developed an MOF-derived CuO/rGO heterostructure composite with a p-p type heterostructure. Through the synergistic effect of the p-p heterojunction, the sensitivity of the sensing material toward CO_2_ was effectively improved. The results showed that the composite sensing material with 5 wt% rGO content exhibited the best performance: at room temperature (25 °C), its response value toward 500 ppm CO_2_ was 39.6, which was much higher than that of pure CuO and pure rGO. It also had long-term stability, making it a high-performance sensor capable of CO_2_ sensing at room temperature.

**Figure 17:**
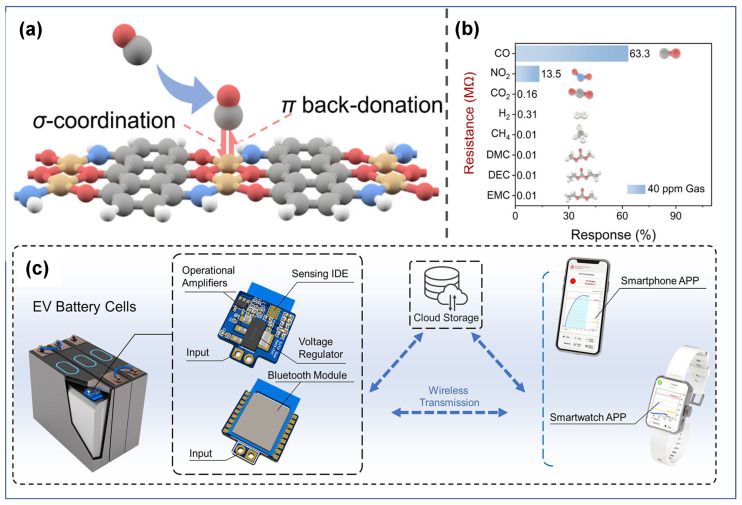
(**a**) Sensing mechanism for 1D Cu_2_DADHA-based MOF materials and (**b**) sensor selectivity for CO and (**c**) gas concentration detection module for monitoring TR gases [112].

Research on CO gas sensing for TR monitoring is also scarce. In 2025, Liu et al. [129] developed a sensor for detecting CO gas generated during LIB TR, as illustrated in Figure 17a. This sensor uses a thin-film material made of a novel one-dimensional (1D) conductive metal–organic framework (Cu_2_DADHA) as the sensing material. Its sensing mechanism relies on the Lewis acid–base interaction between CO molecules and Cu sites, which endows the sensor with reliable gas detection capability. It exhibits excellent room-temperature sensing performance, with high sensitivity and selectivity toward CO, and an ultra-low detection limit of 235 ppb. It can work under oxygen-free and water-free conditions and has long-term stability. As shown in Figure 17c, they also developed an integrated sensor module and designed a simulation test for real-time monitoring of CO concentration, enabling multi-level alarms at different concentrations.

Although few studies have been conducted on CO sensor materials directly targeting TR, there are still numerous CO sensors with potential applications in the field of TR. For instance, Wang et al. [130] adopted a metal doping strategy and successfully synthesized Pt/ZnO nanosheets via a simple one-pot hydrothermal method and calcination treatment for CO gas sensing. The results showed that the introduction of Pt nanoparticles significantly improved the gas-sensing properties of the sensing material and reduced the operating temperature. Pt nanoparticles enhanced CO sensing performance through the combination of chemical sensitization and electronic sensitization. The Pt/ZnO-based gas sensor with the optimal loading ratio exhibited high selectivity and high sensitivity toward CO: it showed a good response toward CO at 180 °C, with fast response/recovery times (6 s/19 s) and a detection limit as low as 0.1 ppm. Hu et al. [131] designed and prepared a CuO-loaded In_2_O_3_/CeO_2_ heterojunction nanofiber, where CuO was dispersed on the surface of the fiber structure. The formation of the heterojunction improved the sensor performance: the sensor could operate at a relatively low temperature of 70 °C, showed strong responsiveness and selectivity toward CO gas, and realized the detection of trace CO gas with an excellent detection limit as low as 50 ppb.

### 6.5. Research Progress of Gas-Sensitive Materials for Multiple TR Gases

Due to the complexity of gas production during LIB TR, in addition to sensors targeting a single gas, many studies have focused on developing sensors with adjustable selectivity or sensitivity to multiple gases.

**Figure 18:**
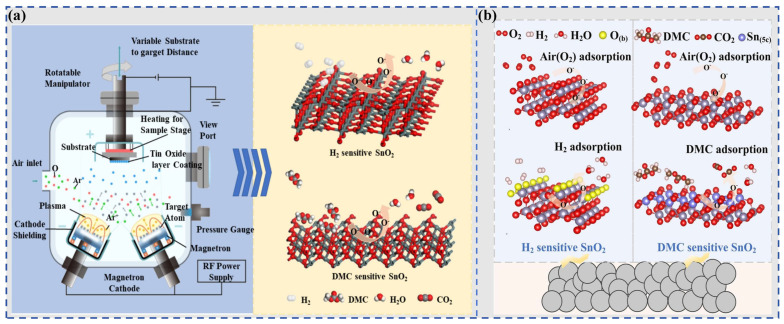
(**a**) SnO_2_ with different crystal planes and gas selectivity was prepared using reactive magnetron sputtering and (**b**) the sensing mechanism of SnO_2_ on DMC and H_2_ gases with different exposure surfaces [132].

In 2025, Shen et al. [132] proposed an innovative strategy to modify gas selectivity by regulating the proportion of exposed crystal planes of SnO_2_. They prepared two SnO_2_ sensors with high sensitivity to H_2_ and DMC via magnetron sputtering technology, as illustrated in Figure 18. By adjusting the oxygen–argon ratio and substrate temperature during the sputtering process, they controlled the exposed crystal planes of the SnO_2_ (110) crystal plane. It had a response value of 808% toward 100 ppm H_2_, but only 209% toward DMC of the same concentration. The SnO_2_ prepared under low oxygen flow rate and a substrate temperature of 0 °C was dominated by the (101) crystal plane. It showed a response of 752% toward 100 ppm DMC, but only 216% toward H_2_. Both sensors exhibited excellent selectivity and ultra-low detection limits (12 ppb for H_2_ and 18 ppb for DMC), and could maintain stable responses within 30 days. In the lithium-ion battery overcharge TR simulation experiment, the two sensors effectively distinguished gas components and could issue a warning 434 s before TR, verifying their application potential.

Zhang et al. [133] used uio66-MOF material as a precursor to prepare Fe_3_O_4_@uio66 core–shell composites. Owing to the pre-concentration effect and thermal stability of the uio-66 shell, the sensor exhibited good selectivity toward VOCs such as DMC, DEC, ethanol, and formaldehyde, as well as long-term aging stability. In the simulated leakage test of the LIB electrolyte, the sensor could detect electrolyte leakage within 30 s. This provides a potential application for the safety monitoring of LIBs.

Gao et al. [121] prepared CsPbBr_3_@In thin-film gas-sensitive materials to realize the detection of electrolyte vapor at room temperature, where the coordination of indium acetate improved the gas-sensing properties. At room temperature, the sensor showed good responsiveness toward gases, including EMC, DEC, and ED, along with fast response capability and long-term stability. To effectively identify the components and concentrations in gas mixtures, they also combined the sensor with a deep residual network (a type of artificial intelligence algorithm). During hydrodynamic simulation detection, the implanted sensor only required 3.1 s to generate a response.

## 7. Conclusions and Outlook

This paper has discussed and summarized the common gases in the field of TR monitoring for lithium-ion batteries, and clearly identifies H_2_, electrolyte vapors (DMC, EMC, etc.), CO_2_, CO, and CH_4_ as the core target gases for warning of TR. In addition, we have detailed the research progress and applications of semiconductor sensors for these types of gases in the field of TR monitoring, aiming to provide support for the research on TR monitoring and warning of LIBs, as well as gas sensing. Currently, most studies enhance gas-sensing performance through methods such as metal doping modification (e.g., Ag, Pt, Pd), regulation of material morphology and structure (e.g., 2D nanosheets, 3D network structures, flower-like structures, cubic structures), and construction of heterostructures in composite materials (e.g., amorphous heterojunctions, ternary heterojunctions).

We believe that there is still room for further development of semiconductor sensors in this field, and future research may focus on the following aspects:

1. Research on warning gases for different batteries needs to be further refined. Variations in battery type, TR initiation method, battery exhaust activation time, and sensor detection limit may all lead to differences in the characteristics of TR-generated gases and the concentration distribution of characteristic gases. Therefore, it is necessary to conduct targeted research on specific TR scenarios and screen for the most appropriate monitoring targets to ensure the accuracy of warnings. Additionally, the impact of the battery’s surface temperature on the sensor during venting should be considered, and it is even more critical to account for the issues arising from internal temperature variations when integrating sensors internally.
2. Higher design requirements for semiconductor sensors in TR gas monitoring. Existing studies inevitably have certain limitations. For instance, some semiconductor materials suffer from issues such as excessively high operating temperatures, insufficient long-term stability, baseline drift, and prolonged response time. This needs further improvement to better align with the needs of TR warning applications. This requires the development of sensors capable of operating at lower temperatures to reduce power consumption while avoiding safety hazards caused by excessive heat. Additionally, improving the long-term stability of sensing materials is critical for achieving continuous monitoring of battery safety status, and minimizing sensor size is necessary to better adapt to battery management systems. Furthermore, higher requirements are placed on the response speed and sensitivity of semiconductor sensors: faster and more sensitive responses provide ample time for TR warning and remediation. Therefore, further optimization of sensing materials is required to improve their gas-sensing performance.
3. Challenges in integrating gas sensors into battery management systems. Currently, TR monitoring relies on the gas release behavior of batteries, restricting its applicability to battery types that exhibit gas release during TR, including those equipped with safety valves (which open to release gas) and pouch cells (which expand and rupture to expel gas). This external signal-based monitoring method lacks sufficient temporal advantages. To address this issue, small sensors can be embedded inside batteries to monitor internal gas release status. While this significantly enhances TR warning capabilities, it inevitably increases costs. Furthermore, MOS materials are prone to cross-sensitivity to different gases in complex environments, potentially leading to false alarms. To mitigate this, a single sensor can be combined with machine learning algorithms (e.g., Random Forest, RF) or other artificial intelligence algorithms to construct a hybrid gas concentration prediction model. This model enables graded concentration early warning and sensitive detection of multiple gases, avoiding false alarms through cross-validation of multi-gas sensing data. Additionally, the integrated application of multiple sensors (e.g., combining gas sensing with voltage and current monitoring) can improve monitoring efficiency and performance, comprehensively capture battery operating status, and overcome the inherent limitations of single-parameter monitoring.

Despite the limited widespread application of semiconductor sensing materials for gas monitoring in TR warning to date, they have demonstrated significant potential. With continuous advancements in material modification and device integration technologies, such semiconductor sensors are expected to play an important role in the TR warning of lithium-ion batteries.

## Figures and Tables

**Table 1:** Basic characteristics of exhaust gases generated during the TR process.

Temperature (°C)	Venting Gases	Poisonousness	Flammability and Explosiveness
70–90	C_2_H_4_	low toxicity, but anesthesia at high concentrations	explosion limits (2.7–36%)
	CO_2_	non-toxic	non-flammable
	O_2_	non-toxic	combustion-supporting
120–140	C_3_H_6_	anesthesia at high concentrations	explosion limits (2.4–10.3%)
	C_2_H_4_	anesthesia at high concentrations	explosion limits (2.7–36%)
	CH_4_	non-toxic	explosion limits (5–15%)
170	O_2_	non-toxic	combustion-supporting
	HF	highly toxic (LC_50_ *: 130 ppm/1 h) and strongly corrosive	non-flammable
	PF_5_	toxic and highly corrosive	non-flammable
	C_2_H_4_	low toxicity, but anesthesia at high concentrations	explosion limits (2.7–36%)
200	CO	asphyxiation at high concentrations (LC_50_: 1800 ppm/1 h)	explosion limits (12.5–74.2%)
	CO_2_	non-toxic	non-flammable
	CH_3_F	anesthesia at high concentrations	22.2% (upper explosive level)
300	H_2_	non-toxic	explosion limits (4–75%)
	HF	highly toxic (LC_50_: 130 ppm/1 h) and strongly corrosive	non-flammable

* The toxicity level refers to the Chemical Classification and Labeling Standard Part 18: Acute Toxicity (GB/T 20577-2006). LC_50_ is the median lethal concentration for rats via inhalation. Data are sourced from authoritative chemical safety databases (MSDS) and relevant literature reports.

**Table 2:** Summary of GC analyses of vented gases in different batteries [66].

Battery Chemistry	H_2_ (%)	CO (%)	CO_2_ (%)	CH_4_ (%)	C_2_H_2_ (%)	C_2_H_4_ (%)	C_2_H_6_ (%)
**LFP**	24.34	4.5	25.39	5.9	0.08	3.26	1.29
**LTO**	8.41	5.3	37.6	1.23	0.0008	1.38	0.40
**NCM 1**	12.39	30.30	13.22	10.50	0.0026	0.10	0.16
**NCM 2**	12.54	28.06	19.91	12.90	0.0027	0.16	0.21

**Table 3:** Summary of Warning Times Based on Gas Detection in Several Battery TR Monitoring Studies.

Battery Type	TR Trigger Method	Criteria for Determining the Start of TR	Total Reserved Warning Time	Ref.
11.6 Ah NMC pouch cell	OT (heating at 5 °C/min)	The rate of temperature rise is accelerating or voltage drop	VOC: 7.1–17.3 min	[87]
	OC (1 C)		VOC: 6.3–8.5 min	
20 Ah LFP prismatic battery	OC (0.5 C)	After a sudden voltage drop, the temperature rises sharply, accompanied by an internal short circuit	H_2_: 5–6.7 min; VOC: 3.3 min; CO: 1.7 min	[89]
40 Ah LFP prismatic battery	OC (1–6 C)	The temperature rise rate reaches its peak	0.6–7 min	[88]
NCA 18650 cylindrical battery	OT (fixed plate temperature 250–500 °C)	The temperature rise rate reaches its peak	0.2–7.2 min	[90]
6 Ah and 67 Ah LFP pouch cell	OT (500 W heating)	The rate of temperature rise (dT/dt) exceeds 1 °C/s	2 min (6 Ah) * 12.7 min (67 Ah) *	[91]
750 Wh NCM pouch battery (3 × 60 Ah in parallel)	OT (heating at 7 °C/min)	Voltage drop	4.4–14 min	[92]
32 and 50 Ah LFP prismatic battery	OT (500 W heating)	The rate of temperature rise (dT/dt) exceeds 1 °C/s	Around 0.7–7 min (32 Ah) Around 1.8–7.5 min (50 Ah)	[85]

* Time starts logging from battery venting.

**Table 4:** Summary of the performance of DMC sensing materials.

Material	Target Gas	Conc. (ppm)	Response	T (°C)	Res/Rec. (s)	LOD (ppb)	Ref.
Bi_2_O_3_	DMC	100	295.7%	160	44/34 (10 ppm)	50	[75]
Ag@Ag_2_O-SnO_2_	DMC	100	106	200	28/55 (100 ppm)	11.76	[109]
Nd-SnO_2_	DMC	50	38.13	150	137/463 (1 ppm)	20	[110]
Co/Pd-SnO_2_	DMC	50	~22	150	66/240 (1 ppm)	500	[111]
Cs_2_SnCl_6_	DMC	100	7.05	200	82/83 (20 ppm)	/	[79]

**Table 6:** Summary of the performance of EMC sensing materials.

Material	Target Gas	Conc. (ppm)	Response	T (°C)	Res/Rec. (s)	LOD (ppb)	Ref.
Pd-WO_3_	EMC	10	17.8	275	19/860	100	[116]
SnO_2_	EMC	20	32.46	140	71/257 (1 ppm)	10	[117]
SnO_2_	EMC	10	7.24	140	100/930	160	[118]
SnO_2_	EMC	10	7.95	210	14/17	500	[119]
MnSnO_3-x_/NiO	EMC	10	93.8%	180	34/204	200	[120]
CsPbBr_3_@In	EMC	1500	0.21	RT	34/41	10 (ppm)	[121]

## Data Availability

No new data were created or analyzed in this study.
